# Biological study of skin wound treated with Alginate/Carboxymethyl cellulose/chorion membrane, diopside nanoparticles, and Botox A

**DOI:** 10.1038/s41536-024-00354-2

**Published:** 2024-02-27

**Authors:** Naimeh Mahheidari, Mohammad Kamalabadi-Farahani, Mohammad Reza Nourani, Amir Atashi, Morteza Alizadeh, Niloofar Aldaghi, Majid Salehi

**Affiliations:** 1 Student Research Committee, School of Medicine, Shahroud University of Medical Sciences, Shahroud, 3614773955 Iran; 2 Department of Tissue Engineering, School of Medicine, Shahroud University of Medical Sciences, Shahroud, 3614773955 Iran; 3 Tissue Engineering and Regenerative Medicine Research Center, Baqiyatallah University of Medical Sciences, Tehran, 1435916471 Iran; 4 Department of Dental and Biomedical Materials Science, School of Dentistry, Nagasaki University, Nagasaki, 8528102 Japan; 5 Tissue Engineering and stem cells research center, Shahroud University of Medical Sciences, Shahroud, 3614773955 Iran; 6 Department of Hematology, School of Allied Medical Sciences, Shahroud University of Medical Sciences, Shahroud, 3614773955 Iran; 7 Health Technology Incubator Center, Shahroud University of Medical Sciences, Shahroud, 3614773955 Iran

**Keywords:** Experimental models of disease, Regenerative medicine

## Abstract

A hydrogel-based wound dressing with desirable properties is necessary for achieving functional skin integrity post-injury. This study focuses on preparing a hydrogel using Alginate/Carboxymethyl cellulose (Alg/CMC) as a base material. To evaluate its regenerative effects on full-thickness wounds, diopside nanoparticles and Botulinum toxin A (BTX-A) were incorporated into the hydrogel along with chorion membrane. The diopside nanoparticles (DNPs) act as a proangiogenic factor, promoting proliferation and regulating inflammation, while the chorion membrane facilitates these processes. Additionally, BTX-A prevents scar formation and aids in wound closure. The nanoparticles and hydrogel were characterized using various techniques, and their cytocompatibility was assessed. In vivo studies and quantitative polymerase chain reaction analysis showed that wound area reduction was significant after two weeks of treatment with the Alg/CMC/ChNPs/DNPs/BTX-A hydrogel. Overall, this scaffold demonstrated potential for promoting tissue regeneration and new epithelization formation, making it a promising candidate for enhancing skin restoration in wound treatments.

## Introduction

Skin is a multilayered supportive structure and acts as a strong barrier following external injury unless the integrity of the different skin layers becomes extremely disturbed^1^. Furthermore, skin is always highly susceptible to different types of damage. skin tissue injury can be further subdivided into acute and chronic unless otherwise specified to the depth as either partial or full-thickness wounds^2^. Chronic wounds require an appropriate treatment to progress through the healing process successfully leading to a scar-free tissue repair. Previous studies reported that failure in full-thickness tissue regeneration leads to a hypertrophic scar^3,4^.

Skin injury improvement and tissue regeneration are achieved through a well-organized four dynamic wound healing process including hemostasis, inflammation, proliferation, and remodeling steps in a timely manner, overall, it leading accelerates damaged tissue recovery. In addition, adequate angiogenesis and re-epithelization often contribute to growth factors releasing and contribute to wound closure^5,6^.To provide an ideal dressing, suitable characterization should be considered including maintaining a high humidity at the wound bed while absorbing excess exudate. In addition, a suitable physiochemical membrane at the injury surface facilitates cell migration, adhesion, and proliferation. Thus, a proper wound dressing should possess a highly porous structure, desired degradation rate, and minimal cytotoxicity effect besides high biocompatibility to reduce immunogenicity^6,7^. Meanwhile, there are various marketed wound care products to use in clinical application, effective wound treatment to recover skin function following chronic and severe skin damage still needs serious medical interventions, thus, a proper wound dressing is highly important to design to induce neovascular genesis and neocollagenesis besides restoration of skin integrity and function^8,9^.

Previous studies demonstrated that natural polymers are excellent candidates and satisfy all essential requirements and favorable micro-environment for ideal wound dressing rather than the other conventional materials^10,11^. Functional and crosslinked polymeric hydrogels are extensively developed in recent years to assist wound healing because of their eligible properties and physiochemical similarities to the extracellular matrix. Interestingly they are useful as a carrier for therapeutic agents or controllably sustainable release of nanoparticles^12,13^. A recent study indicated that nanocomposites based on hydrogel can significantly promote the wound-healing process^14^.

Moreover, chorion and amnion membranes are highly rich in protein matrix and facilitate cell migration at the wound area. They also possess anti-inflammatory, antibacterial, biodegradable, and low immunogenic properties. Also, the chorion membrane has a well-tolerated and thicker structure, and higher reservoirs of growth factors and cytokines than that of the amnion membrane. Although the amnion membrane is well-known as anti-fibrosis, the chorion membrane is introduced as a more suitable membrane to reduce inflammatory reactions in tissue repair. Thus, they are well incorporated into the wound bed and facilitate full-thickness wound healing by accelerating re-epithelialization and granulation besides reduced scar formation with no need for wound dressing changes, daily^15–19^. Furthermore, the promising wound healing effect of decellularized membranes such as acellular dermal matrix (ADM), AlloDerm, GraftJacket, and decellularized placenta membranes (amnion and chorion) have been investigated in various researches^20^.

Nanoparticles due to desired antibacterial, proangiogenic, and antiinflammatory properties, play a key role in wound healing process^21,22^. Besides, bioactive nanoparticles such as diopside nanoparticles act as an efficient wound healing agent^23^.

One of the crucial factors of impaired wound healing is poor angiogenesis. Currently, previous studies discovered that diopside, a silicate-based bioactive glass with the chemical composition of CaMgSi2O6 exhibited angiogenic potential in bone regeneration leading to promote rapid tissue repair^24,25^. More importantly, to improve or prevention of scar wounds, it was further found that, Botulinum Toxin Type A not only reduces muscle tension but also induces chemo-immobilization at the wound bed so that tension decreased resulting in successful wound healing^26,27^.

In this study, we fabricated a hydrogel-based wound dressing containing Botox A, chorion, and diopside nanoparticles for full-thickness wound treatments. Therefore, the main purpose of the present study is to develop a potential wound dressing to improve skin restoration.

## Results

### Characterization of the decellularized human chorion membrane

**Fig. 1:**
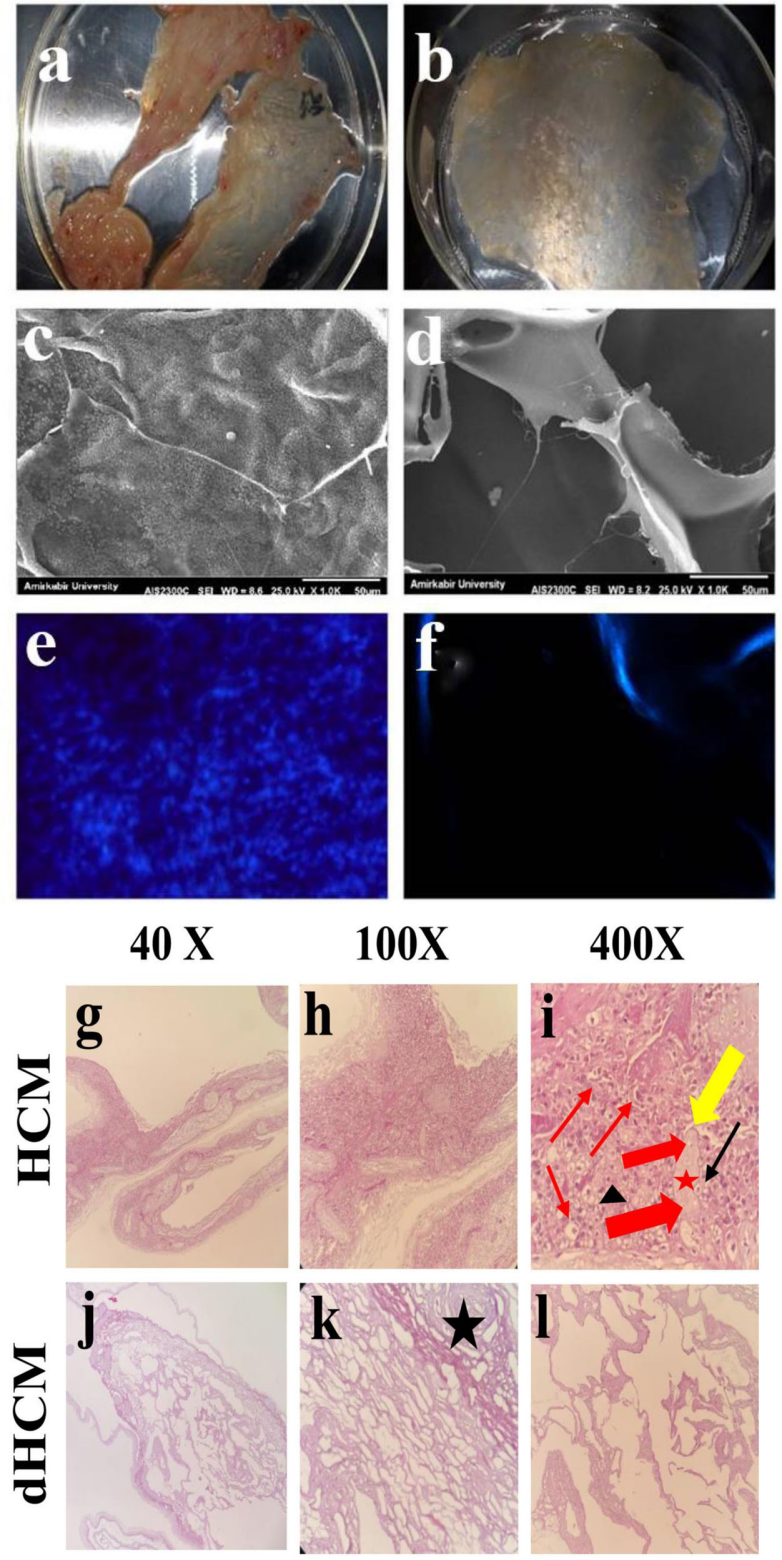
Characterization of HCM and dHCM. Representative photographs of native human chorion membrane (HCM) (**a**) and decellularized human chorion membrane (**b**). SEM image of HCM (**c**) and dHCM (**d**), DAPI staining of intact chorion membrane (**e**) and acellular chorion membrane (**f**). Histology characterization of an intact human chorion membrane (**g**–**i**) and dHCM (**j**–**l**). Hofbauer cell was shown by a black thin arrow, whereas blood vessels, fetal blood vessels, and syncytial trophoblast were shown by a red thin arrow, red thick arrow, and yellow thin arrow respectively. On the other side, black star and black arrowhead are used to label the connective tissue and intervillous space, respectively.

**Fig. 2:**
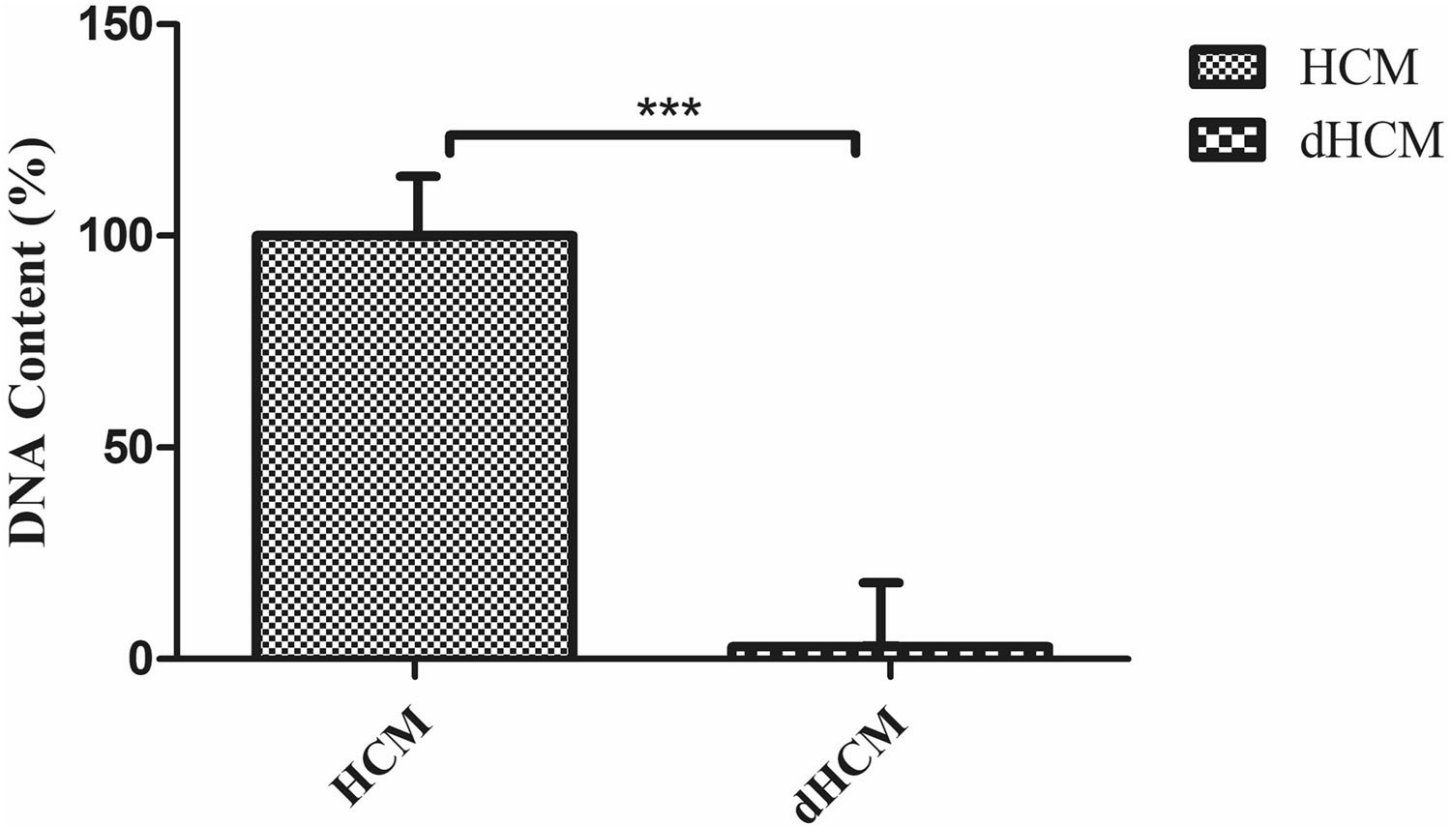
DNA content in HCM and dHCM. Double-stranded DNA (dsDNA) quantification is reported in percent ***p ≤ 0.001.

The efficiency of the decellularization assay was investigated and the results are summarized in Fig. 1. The significant differences in tissue structure between intact human chorion membrane (HCM) and decellularized human chorion membrane (dHCM) were observed. As shown in Fig. 1a, b, a thinner compact layer membrane was obtained after the decellularization protocol compared to the native membrane. SEM images exhibited that the dHCM structure is highly preserved after treatments (Fig. 1c, d). Furthermore, no obvious cell structure was detected in dHCM related to native HCM. According to the results obtained from DAPI staining assay, no nuclei were observed in the acellular chorion membrane as shown in Fig. 1e, f. Nevertheless, the pale blue layer was observed in the acellular chorion membrane attributed to autofluorescent ECM components such as collagen in membrane structure representing^28^ its high stability after physical and chemical protocols used in the decellularization process. Cellular removal results were also corroborated by H & E staining (Fig. 1j–l). Moreover, H & E staining of HCM and dHCM was in accordance with the result of DAPI staining, representing no nuclei in dHCM compared to HCM. To study the structure of dHCM and HCM, H and E staining was performed. Figure 1j–l demonstrated that this treatment was successful in removing cells from HCM. After decellularization protocol, approximately all of the epithelial and mesenchymal cells were eliminated, however, the other components of the extracellular matrix in dHCM structure remained intact. Chorion villi, trophoblast cells, chorion mesenchymal cells, basal membrane, collagen fibers, connective tissue, and Hofbauer cells were presented in the membrane in Fig. 1g–i. Conversely, in the dHCM structure, no cells were observed, (Fig. 1j–l) Hofbauer cell was shown by a black thin arrow, whereas blood vessels, fetal blood vessels, and syncytial trophoblast were shown by a red thin arrow, red thick arrow, and yellow thin arrow respectively. On the other side black star and black arrow head are used to label the connective tissue and intervillous space respectively. Figure 2 represents the DNA amount quantification analysis in HCM and dHCM. In summary, the percentage of DNA content exhibited a significant decrease in dHCM and the mean was reported 2.96 ± 0.61compared to intact human chorion membrane, a *p*-value less than 0.001

### Evaluation of the ChNPs and DNPs

**Fig. 3:**
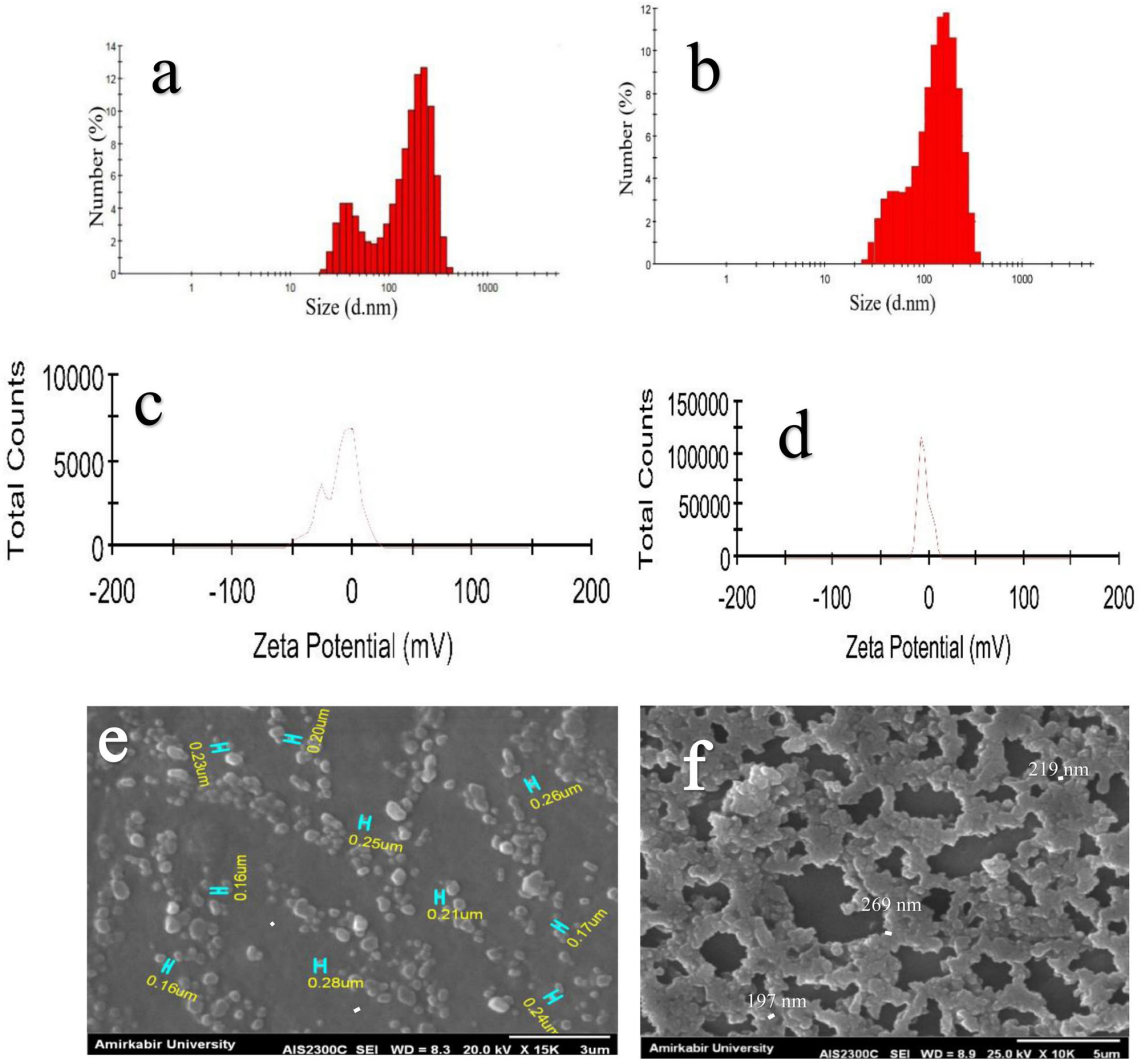
Characterization of Chorion and diopside nanoparticles. Size measurements of (**a**) Chorion nanoparticles and (**b**) diopside nanoparticles. Zeta potential of (**c**) chorion nanoparticles and (**d**) diopside nanoparticles. FE-SEM image of (**e**) Chorion nanoparticles and (**f**) diopside nanoparticles.

The average hydrodynamic diameters of ChNPs and DNPs are determined 350 nm and 147 nm with PDI of 0.5 and 0.4 respectively. Differences were obtained statistically significant at *P* < 0.001. The zeta potential results represent the negative electrical surface charge and stability of ChNPs and DNPs with a mean value of −9.75 ± 14.2 and −4.57 ± 5.97 mV respectively (Fig. 3a–d). Therefore, these particles at this range are considered highly stable and relatively neutral. Figure 3e, f indicates the FE-SEM photomicrographs of chorion nanoparticles and diopside nanoparticles respectively, and investigated the morphology and size of nanoparticles at the 10–15 K magnification. Although the results display diopside nanoparticles aggregation, homogenous distribution is observed by SEM micrograph. The surface of nanoparticles showed both spherical and cubic morphology (Fig. 3f). More importantly, the SEM data correlated with DLS results.

### FTIR spectroscopy

**Fig. 4:**
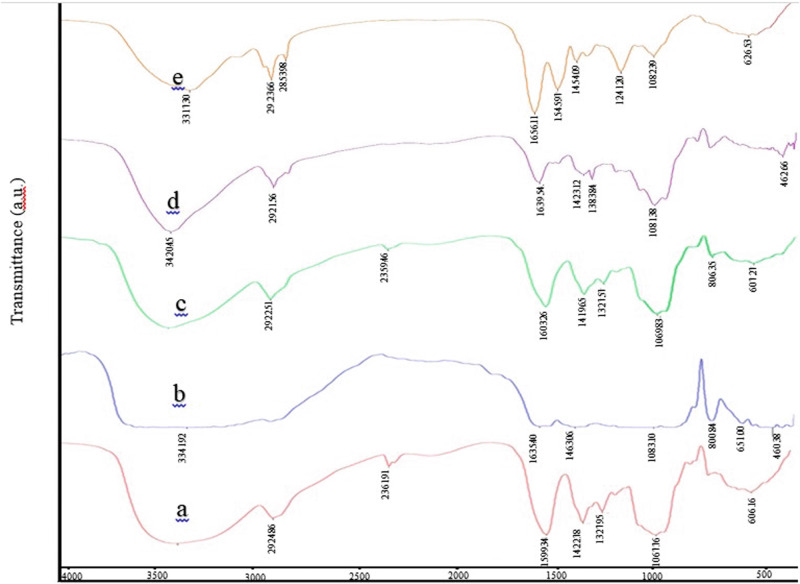
FTIR analysis. FTIR spectroscopy of **a** Alg/CMC hydrogel, **b** Alg/CMC/DNPs, **c** Alg/CMC/BTX-A, **d** Alg/CMC/ChNPs/DNPs/BTX-A, **e** chorion nanoparticles.

Figure 4 demonstrated the ATR-FTIR spectra of Alg/CMC containing DNPs and BTX-A (b, c) in comparison with pure Alg/CMC (a). Based on a previous study^29^ The strong peaks appeared at 3300 cm^−1^, which may be ascribed to the OH vibration band. While low-intensity peaks at 1599, 1422.78, 1327.95, 1067.76 cm^−1^ can be attributed to the asymmetric and symmetric of -COO groups C-O and C-O-C of ester groups of Alg/CMC hydrogel respectively. The weak stretching vibration band around 2924 cm^−1^ and 2361 cm^−1^ are correlated with asymmetric and symmetric C-H in CMC, respectively^29^. Moreover, the strong wide absorption peaks at 606.16 cm^−1^ can be related to the interaction between the Ca ^2+^ cross-linker and carboxyl groups of the structure^30^. Furthermore, according to the literature^31^, in the spectrum of DNPs hydrogels a strong wide stretching mode at 3341.92 cm^−1^ was observed corresponding to hydrogen linkage and small low peaks at 1615.40, 1463.96, 1083.10, 800.84 cm^−1^ were appeared indicating the presence of symmetric stretching of silicate groups and a low-intensity peak at 450.38 cm^−1^ attributed to Mg-O stretching vibration. BTX-A hydrogel structure was confirmed by characteristic peaks at 882.46 cm^−1^ was assigned to twisting vibration bands of –CH2 in toxin structure. The other intensity peaks emerged at similar ranges of Alg/CMC functional groups with a slight shift^32^. Moreover, in Chorion nanoparticles, there are wide range absorption peaks due to the complex components of the chorion structure and various proteins in the structure (Fig. 4e). The band at 1082.39 cm^−1^ probably exhibits the glycol-phospholipids, phospholipids, and the phosphodiester group of nucleic acids, whereas the vibration mode at around 1241.20 cm^−1^ is assigned to the amide III bands of protein. The vibration modes at 626.53 cm^−1^ represent the planar deformation of C=O groups. A small absorption peak at 1454.09 cm^−1^ and two small and sharp modes close to 1545.97 cm^−1^ and 1656.11 cm^−1^ are probably assigned to C–H bending modes, amide II stretching vibration of N–H bending mode and C–N and C=O vibration bands, Meanwhile, two other low peaks at the higher wavenumbers of 2853.98 and 2923.66 cm^−1^ can be assigned to the asymmetric stretching vibration of the CH3 group.

The absorption peaks at 3317.30 cm^−1^ are probably associated with C–H stretching band. Interestingly, in Alg/CMC/ChNPs/DNPs/BTX-A hydrogel, Mg-O groups vibration mode at 462.66 cm^−1^ is obvious (Fig. 4d). A sharp and deep absorption peak at 1081.38 cm^−1^ was observed and can be assigned to both C-O-C of ester groups of Alg/CMC hydrogel and phospholipids of chorion structure. A low absorption peak at 2921.56 cm^−1^ was ascribed to C-H stretching vibration. Also, a low sharp intensity peak appeared at 1383.84 cm^−1^ can be ascribed to C-O groups of Alg/CMC hydrogel.

The Sharp absorption peak at 3420.45 cm^−1^ indicates a highly aligned linkage due to the interaction between the C-H bending mode and highly amount of OH group in hydrogel and also represents the existence of hydrogen bonds. In the present study, all results correlated with the literature.

### Water uptake

**Fig. 5:**
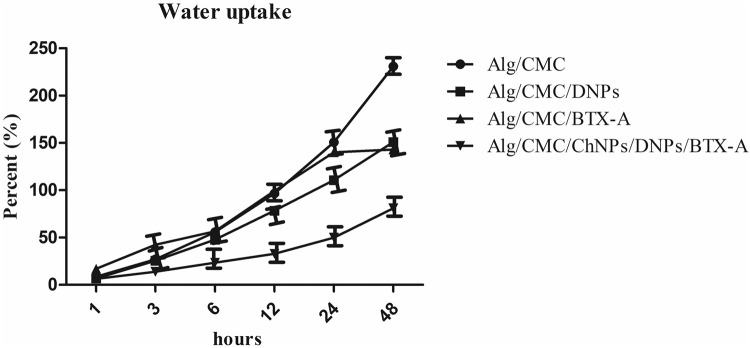
Water absorption percentage at specific hours (1 h, 3 h, 6 h, 12 h, 24 h, and 48 h) in different groups of hydrogels. Data are shown as mean ± SD and reported in cumulative frequency.

The equilibrium absorption capacity of exudates and fluids is a desired property of hydrogel-based materials that exerts a profound influence on wound healing rates^33^. The prepared hydrogel samples were evaluated in PBS solution for 48 h and the results are illustrated in Fig. 5. It is demonstrated that Alg/CMC/DNPs concerning Alg/CMC exhibits a gradual increase at the initial 12 hours, however, the water absorption of 0.1% (w/v) DNPs hydrogel sample reached around 78% and 110% after 12- and 24-h incubation. Finally, the highest absorption was achieved at approximately 150.92%, however, Alg/CMC showed higher water content of 231% After 48 hours. On the other hand, Ag/CMC/BTX-A (200 µl/10 ml) presented a gently increased trend of water absorption Alg/CMC. It exhibited an 89.31%, 99.22%, and 123.44% increase in water uptake capacity after 12, 24, and 48 h respectively. More importantly, after 48 h of incubation in PBS, the structure of BTX-A cannot be investigated owing to hydrogel hydrolysis. Besides, as shown in Fig. 5, Alg/CMC/ChNPs/DNPs/BTX-A showed a gradual and lower increase than the other groups. It begins from 6.39% at the first hour to 80.99% at 48 h after immersion in PBS.

### Weight loss

**Fig. 6:**
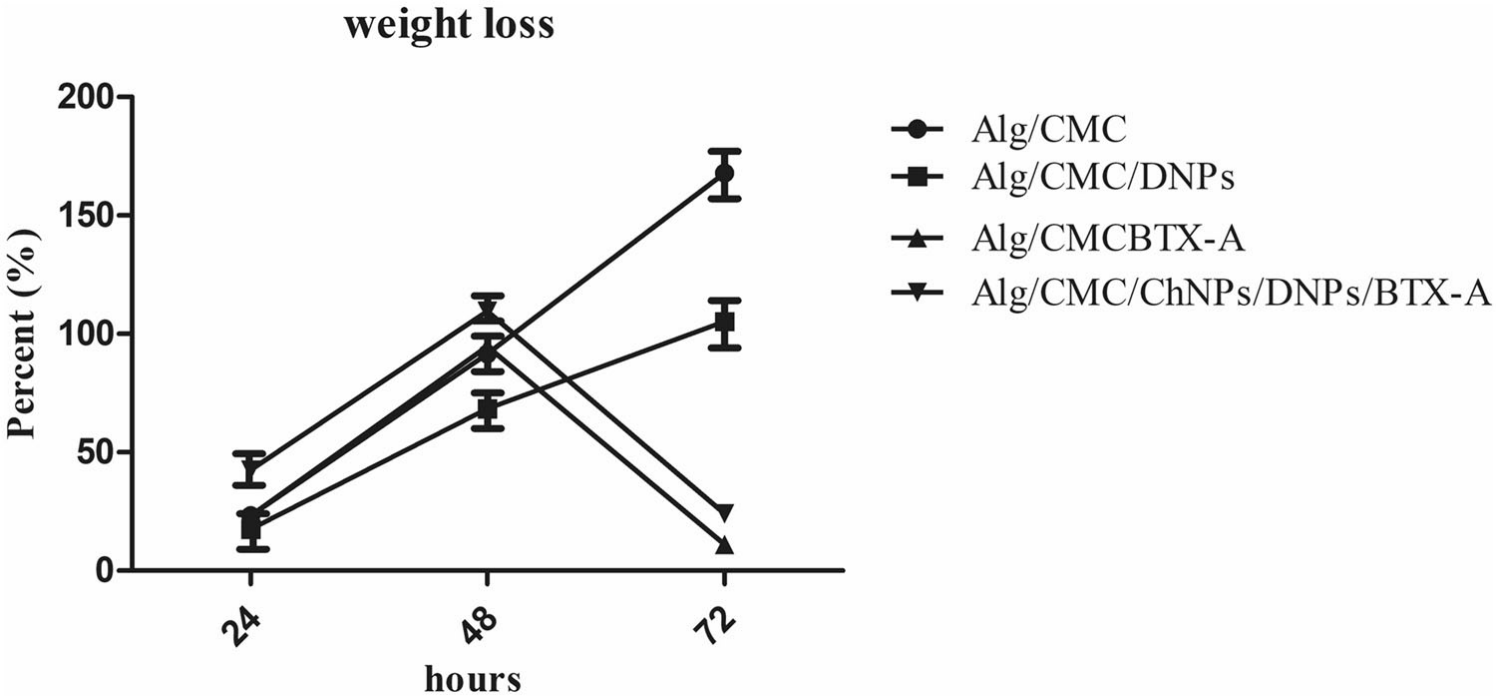
The percentage of the weight loss data at determined hours (24 h, 48 h, and 72 h) was reported for Alg/CMC/ChNPs/DNPs/BTX-A, Alg/CMC/DNPs, and Alg/CMC/BTX-A hydrogels compared to Alg/CMC. Data are shown as mean ± SD and reported.

Weight loss assessment is a critical characterization for hydrogel biodegradability behavior due to the polymer network molecular weight and crosslinked between chains’ increased resistance to hydrolysis in PBS over-determined hours^34^. The degradation rate of Alg/CMC/DNPs and Alg/CMC/BTX-A hydrogels compared to Alg/CMC hydrogel in PBS were studied by weight loss investigation (Fig. 6). The weight loss percentage of Alg/CMC/DNPs increased slowly at about 17.64% and 58.44% over 24 and 48 h of incubation in PBS, respectively. It can be observed that the Alg/CMC/DNPs exhibit a slower degradation rate when compared to Alg/CMC hydrogel.

Besides, Alg/CMC/BTX-A weight loss was impossible to report after 48 h due to poor linkage between Botulinum toxin A and Alg/CMC network in the structure. As expected almost 81.83% of the initial weight of BTX-A hydrogel has been lost which is more than that of Alg/CMC was reported at 61.60% after 48 h. Additionally, Alg/CMC/ChNPs/DNPs/BTX-A showed a sharp increase at 81.31% after 48 h of immersion. Similar to Alg/CMC/BTX-A, after 72 h 90% of hydrogel was dissolved in PBS.

### In vitro Blood Compatibility (BC) Tests

**Fig. 7:**
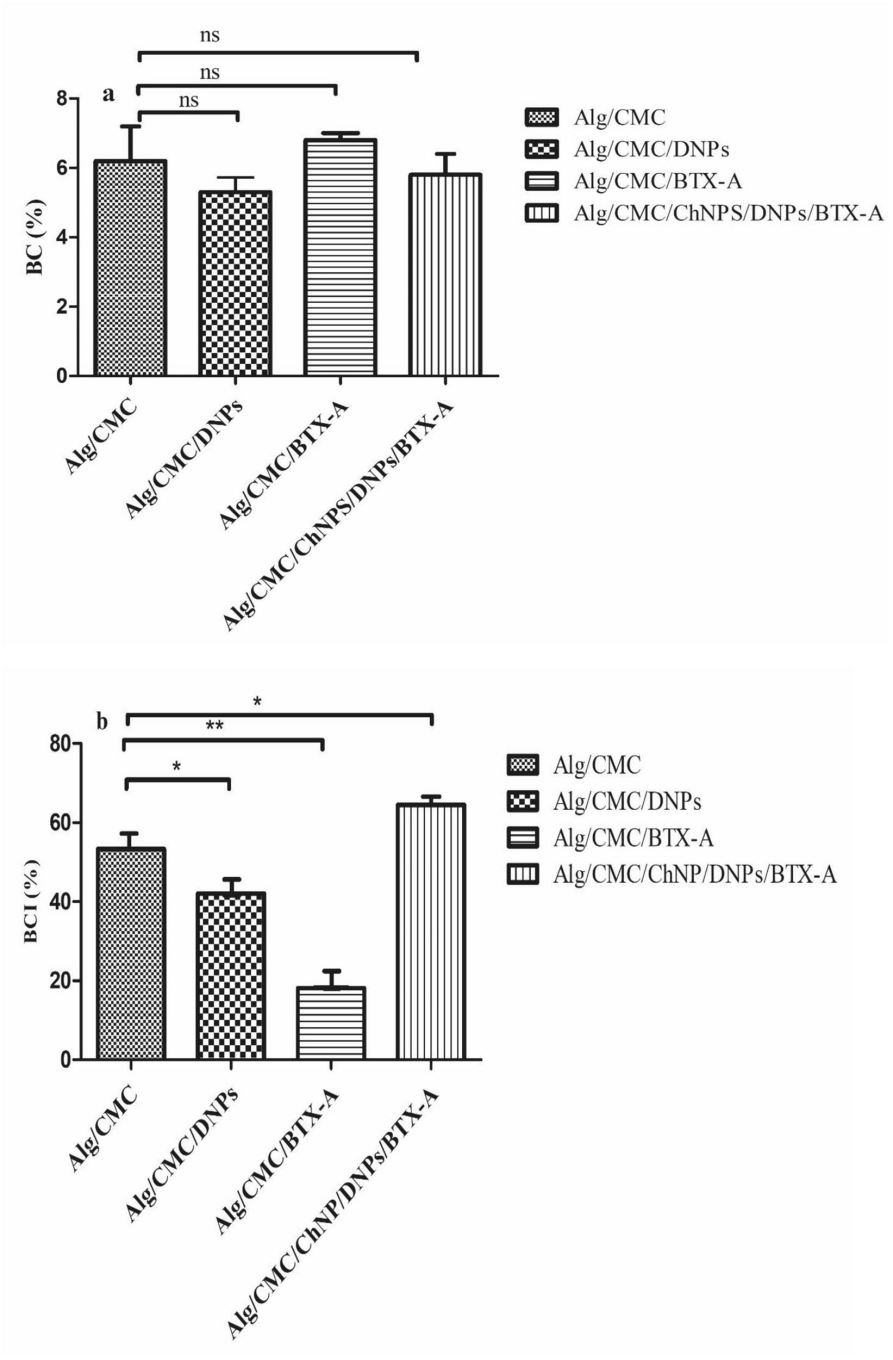
Hemcompatibility tests. Blood compatibility rate (**a**) and Blood Clotting Index (**b**) for Alg/CMC/DNPs, Alg/CMC/BTX-A, and Alg/CMC/ChNPs/DNPs/ BTX-A in comparison with Alg/CMC in vitro, *p* = ns (non-significant), *p < 0.05, and **p < 0.01.

Blood compatibility is an important property for evaluating the hemostatic ability and pro-coagulant activity of fabricated hydrogel samples in favor of the wound healing process^35^. It was investigated by BCI and BC tests. Hemolysis assay or BC of Alg/CMC/DNPs, Alg/CMC/BTX-A, and Alg/CMC/DNP/ChNP-BTX-A were evaluated and compared with Alg/CMC hydrogel. Interestingly, the hemolysis value of the Alg/CMC/BTX-A sample is remarkably higher than that of Alg/CMC/DNPs hydrogel and pure Alg/CMC. Figure 7a displayed that hemolysis percentages of Alg/CMC/BTX-A, Alg/CMC/DNPs, Alg/CMC/DNP/ChNP-BTX-A and Alg/CMC were 6.8, 5.3, 5.8 and 6.2%, respectively. Conversely, DNPs showed a lower hemolysis rate rather than Alg/CMC.

The results indicated that the blood coagulation index (BCI) of Alg/CMC was 53.82% and the BCI rate of Alg/CMC/DNPs, Alg/CMC/BTX-A and Alg/CMC/DNP/ChNP-BTX-A were 41 and 18.2, 66.6% respectively, (Fig. 7b). According to the previous study^36^, a lower BCI rate represents a more excellent pro-coagulation effect of prepared hydrogels. Therefore, the results demonstrate that DNPs and BTX-A improve the excellent coagulation effect (blood clotting effect) of Alg/CMC hydrogel. A slow increase was observed in BCI rate of Alg/CMC/DNP/ChNP-BTX-A sample which might be related to the blood compatibility of chorion nanoparticles.

### In vitro anti-inflammatory activity

#### Inhibition of protein denaturation

**Fig. 8:**
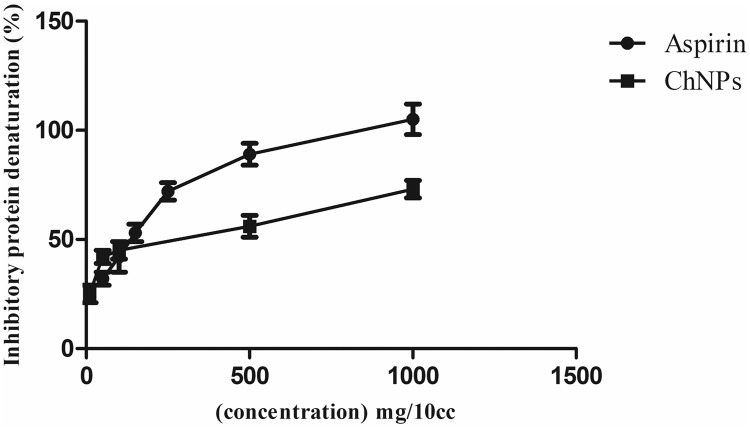
Inhibition of thermally-induced protein denaturation of various concentrations of ChNPs (10, 50, 100, 500, 1000 mg/10 mL) Compared to acetylsalicylic acid (Aspirin) as a reference drug at various concentrations of 5, 10, 15, 25, 50, and 100 mg/mL. *P* value was reported non-significant.

To assess the anti-inflammatory potential of ChNPs, protein denaturation was conducted. The in vitro anti-inflammatory findings of different concentrations of ChNPs compared to acetylsalicylic acid (a standard inflammatory drug) were evaluated against denaturation of BSA (Fig. 8). All concentrations tested showed satisfactory inhibitory potential compared to acetylsalicylic acid. The highest and lowest inhibition percentage of ChNPs against BSA denaturation was recorded at 77% and 25% at concentrations of 1000 mg/10 mL and 10 mg/ml respectively. The reference drug acetylsalicylic acid exhibited inhibitions of 33.3% and 109% at concentrations of 50 and 1000 mg/mL respectively.

#### Tube formation assay (Angiogenesis activity)

**Fig. 9:**
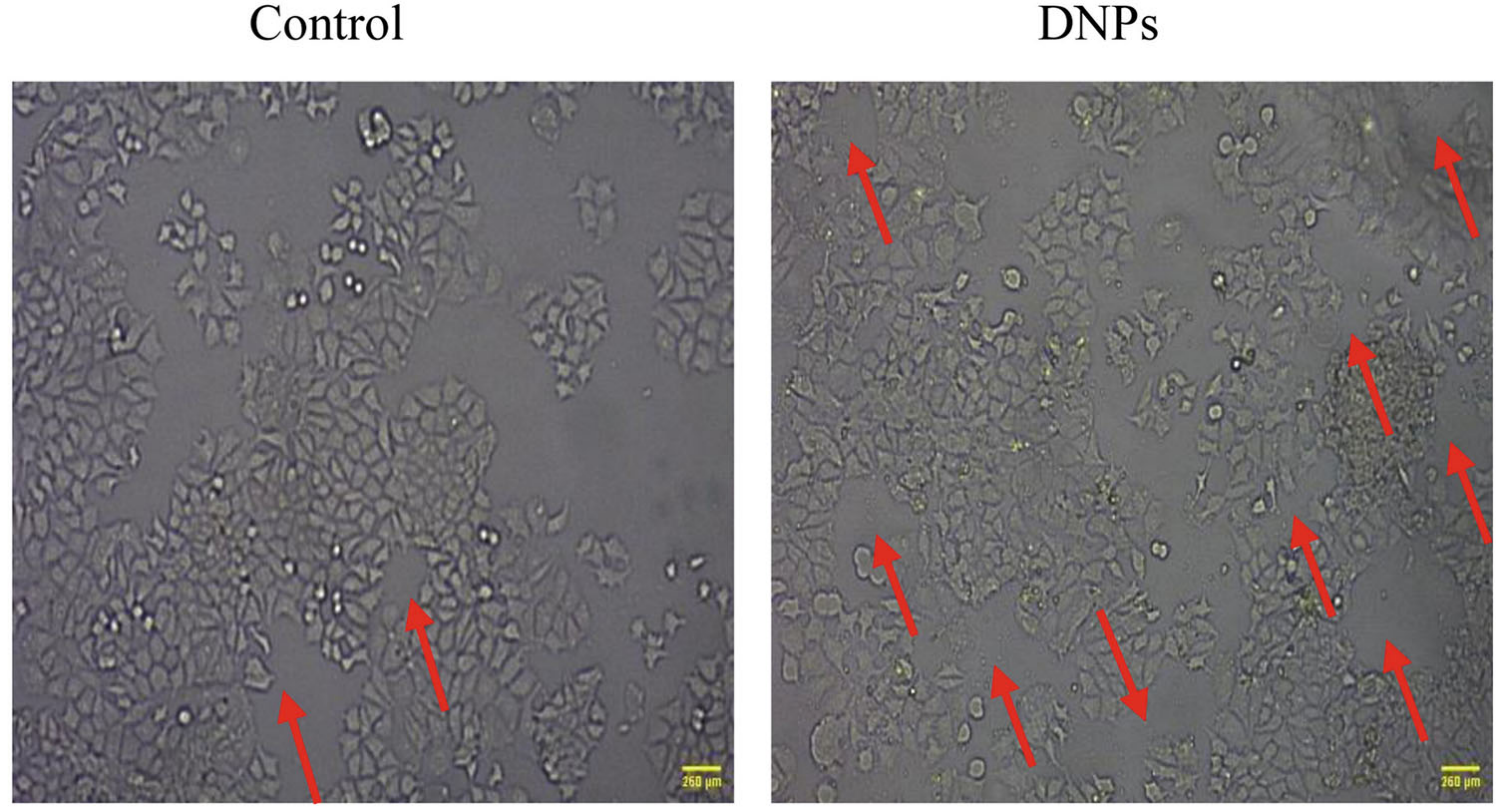
A representative images of tube formation assay for HUVECs seeded on matrigel after treatment with 0.1% DNPs w/v for 12 h in comparison with HUVECs seeded on matrigel as control. The red arrow indicates tubular network formation. Scale bar = 260 µm.

Additionally, a tube formation assay using Matrigel was conducted to study the angiogenesis activity of HUVECs incubated with 0.1% w/v DNPs. As depicted in Fig. 9, after 12 h of incubation, the DNPs group showed a significant improvement, with higher elongated and tube-like structures compared to the control group of HUVECs.

#### Cytotoxicity (MTT assay)

**Fig. 10:**
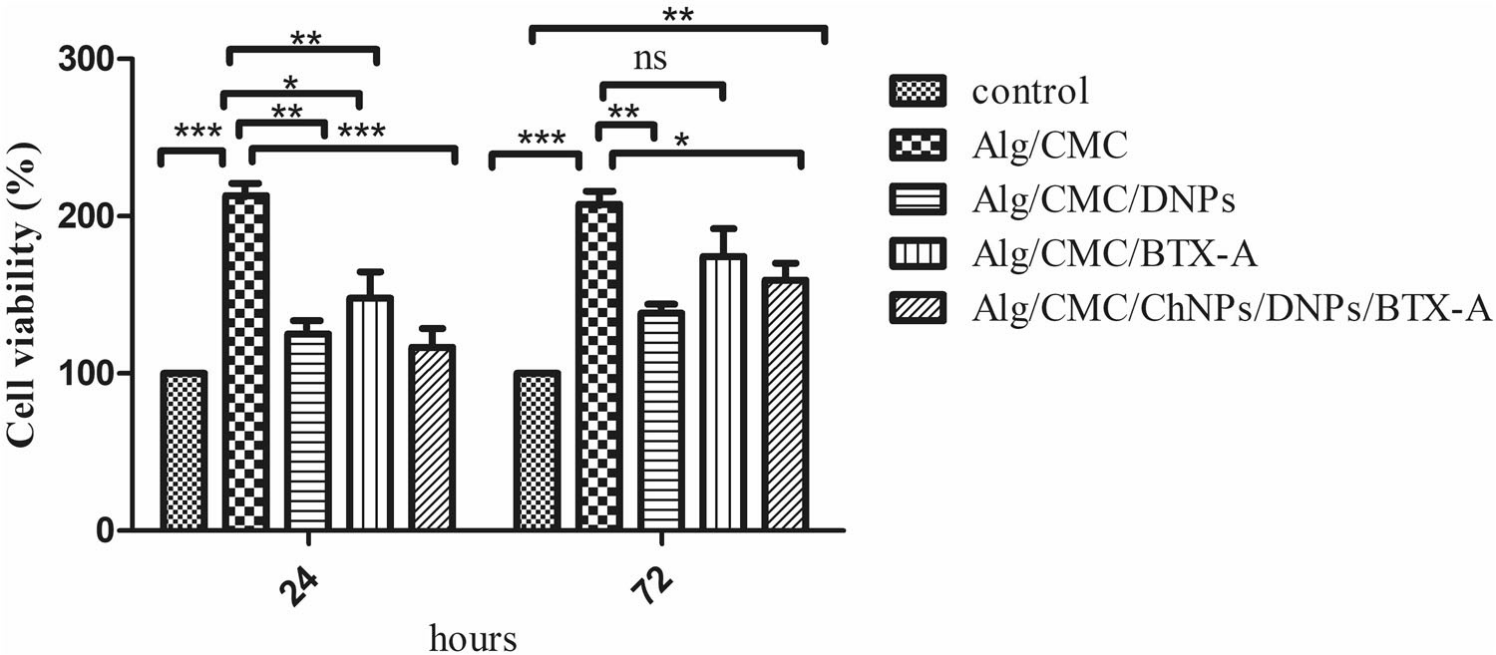
Effects of Alg/CMC containing diopside nanoparticles, Alg/CMC containing Botulinum toxin A, and Alg/CMC containing chorion nanoparticles, diopside nanoparticles, and Botulinum toxin A extracts on 3T3 fibroblast cell line and compared to pure Alg/CMC at 24 and 72 h investigated by MTT assay. Values represent the mean ± standard deviation, ***p < 0.001, **p < 0.01, *p < 0.05 and *P* value = ns (no significant).

The cell viability of Alg/CMC/ChNPs/DNPs/BTX-A, Alg/CMC/DNPs, and ALg/CMC/BTX-A compared to pure Alg/CMC were investigated by indirect MTT assay on a 3T3 fibroblast cell line. According to the results, the hydrogel containing DNPs in comparison to Alg/CMC hydrogel at 24 and 72 h after treatments exhibited a slight increase up to 125% and 138.3% respectively. Furthermore, the results of Alg/CMC/BTX-A cell viability in comparison with Alg/CMC displayed that cell viability percent was slightly higher than that of DNPs samples. Significant cell viability differences between Alg/CMC/DNPs and Alg/CMC/BTX-A in comparison with Alg/CMC were reported as *p* < 0.01 and *p* < 0.05 respectively after 24 h and *p* < 0.01 and non-significant after 72 h respectively. According to the results all samples not only showed no toxicity but also exhibit a positive effect on proliferation rate at both 24 and 72 h after incubation (Fig. 10).

#### In vivo study

**Fig. 11:**
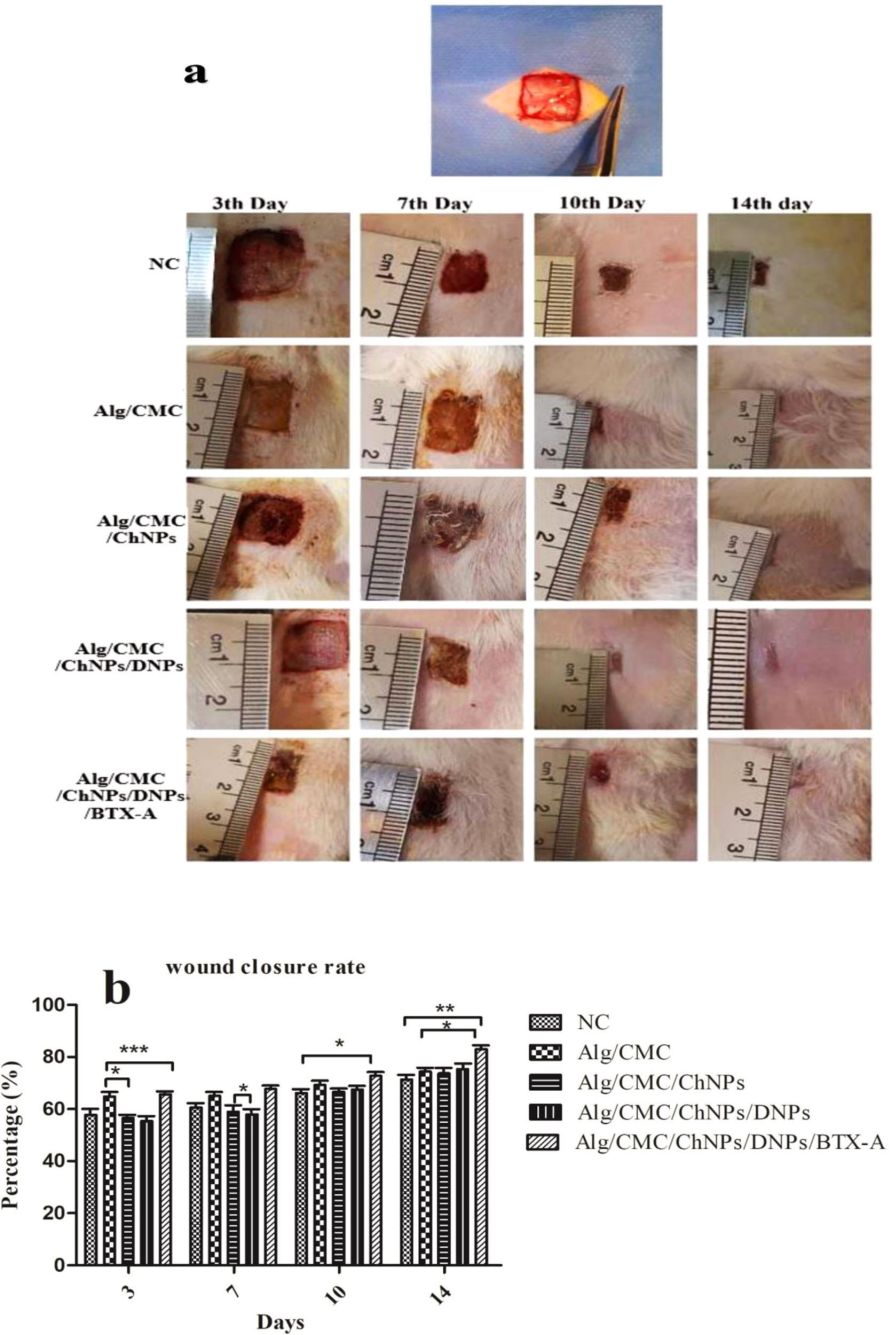
Effect of prepared hydrogel samples on wound healing. **a** Wound healing process observed during 7- and 14-days treatment by NC (Negative control), Alg/CMC, Alg/CMC/ChNPs, Alg/CMC/ChNPs/DNPs, and Alg/CMC/ChNPs/DNPs/BTX-A. **b** Wound closure rates were measured at the 3rd, 7th, 10th, and 14th days. (*n* = 3) *P < 0.0, **P < 0.01, and ***p < 0.001.

The full-thickness skin wound healing process of rat models was monitored over 14 days by digital images from each group at 3^th^, 7^th^, 10^th^, and 14^th^ days (Fig. 11a). Besides, the wound closure rate was measured by wound healing tools software (Fig. 11b). The Macroscopic assessments showed that among all treatment rat models, the groups were treated with Alg/CMC/ChNPs, Alg/CMC/ChNPs/DNPs, and Alg/CMC/ChNPs/DNPs/BTX-A showed remarkably healed and no signs of bacterial infection and scar formation was also observed compared to other rat groups. Furthermore, hair follicle formation can be observed at the wound site on day 14 post-wounding in Alg/CMC/ChNPs /DNPs/BTX-A. Conversely, in the negative control group defect site was not completely healed after two weeks, (Fig. 11a). More importantly, the quantitative assessment of wound closure was measured at the 3^rd^, 7^th^, 10^th^, and 14^th^ days after treatments and compared to control groups and each other. As shown in Fig. 11b wound closure rate results indicated that (smaller wound area than the others) wound area contraction rate was higher in Alg/CMC/ChNPs /DNPs/BTX-A rather than that of the others groups at 3, 7, 10, and 14^th^ days of wound healing with the mean rate of 65.63 ± 7.800, 67.67 ± 7.140, 72.71 ± 6.620 and 82.95 ± 11.69 respectively. Although Alg/CMC/ChNPs and Alg/CMC/ChNPs/DNP groups indicated a slower wound closure rate compared to the control and two other groups on the rd3rd day with an average wound closure rate of 56.57 ± 1.260 and 55.39 ± 2.440 and on 7 days with an average wound closure rate of 58.99 ± 1.540 and 57.97 ± 2.560 respectively, the wound area was significantly decreased in both treatment groups on the 14^th^ day of wound healing and the mean of wound closure rates were reported 73.69 ± 2.425 and 75.28 ± 4.015 respectively on day 14 post-treatment. The mean rate of wound closure in negative control was reported 57.83 ± 6.962, 60.53 ± 4.520, 66.09 ± 3.080, and 71.27 ± 3.105 at the end of the 3^rd^, 7^th^, 10^th,^ and 14^th^ days post-treatment. Differences in Wound closure results were evaluated as statistically significant at *P < 0.0, **P < 0.01, and ***p < 0.001 respectively.

### Histological assessments after 1 and 2 weeks of wound healing

**Fig. 12:**
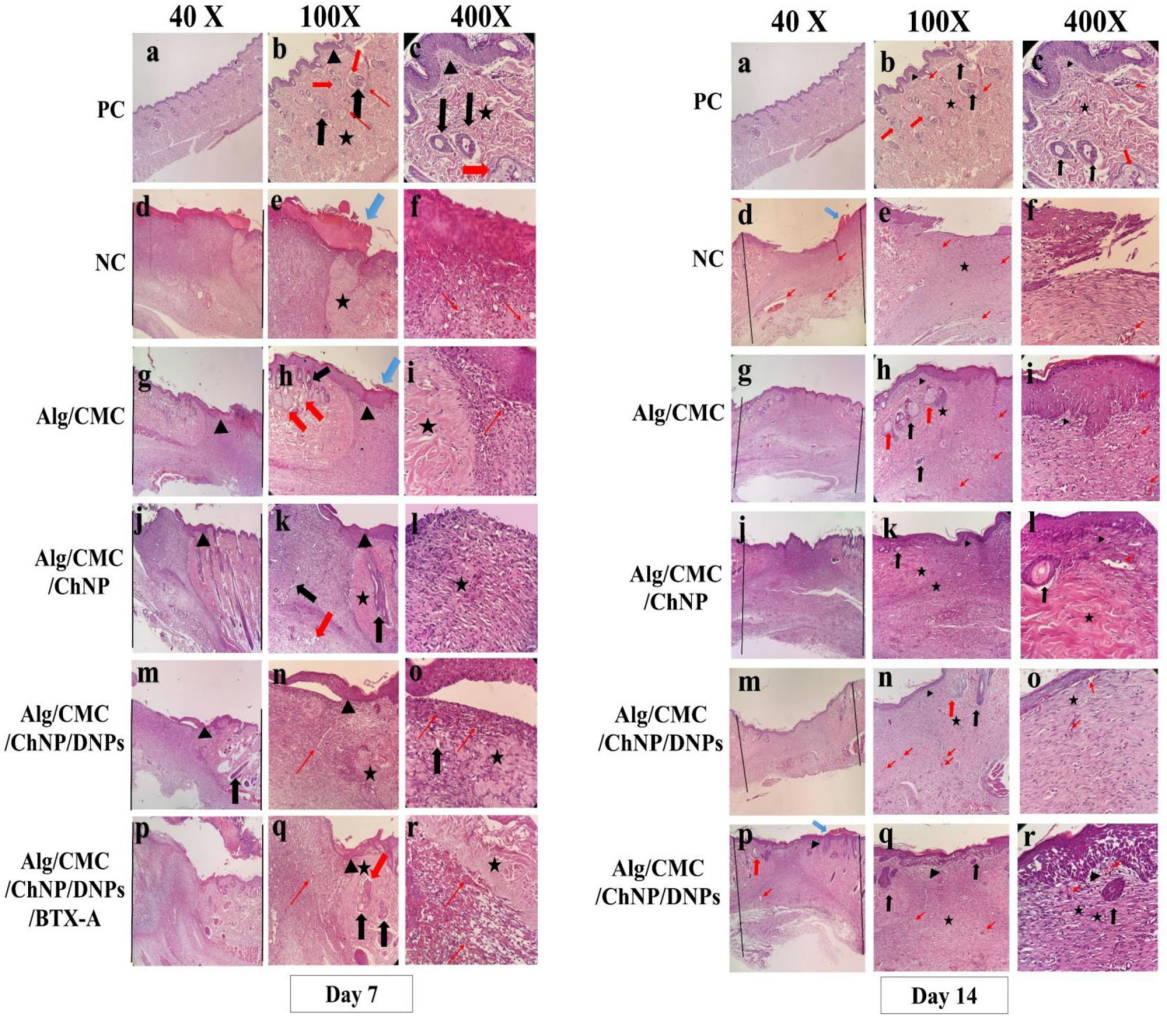
Histopathological study. Hematoxylin and eosin (H&E) staining of the skin of rats in different groups of Alg/CMC (**g**–**i**), Alg/CMC/ChNPs (**j**–**l**), Alg/CMC/ChNPs/DNPs (**m**–**o**), Alg/CMC/ChNPs/BTX-A (**p**–**r**) hydrogel with the comparison of the positive control or PC (**a**–**c**) and negative control (**d**–**f**) after 7^th^ and 14^th^ days of wound healing (Magnification: ×40, ×100, and ×400). The new epithelial formation was shown by (black arrowhead), whereas new blood vessel formation in the dermis was labeled by (thin red arrows) and stars refer to collagen formation, thick black arrow and thick red arrow were used for hair follicles and sebaceous glands, respectively. Blue thick arrow demonstrates crusty scab.

Skin full-thickness wound healing effects of fabricated hydrogels were evaluated on the 7^th^ day and 14^th^ day post-treatments by micrographic photo. The histological results of hematoxylin and eosin-stained slides were investigated in Fig. 12. Also, re-epithelization score was investigated by Image J software. Moreover, new blood vessel scores were measured and compared with normal skin. In Alg/CMC/DNPs (Fig. 12m–o) and Alg/CMC/DNPs/BTX-A (Fig. 12p–r) groups, epithelial layer formation was not obvious on 7 days after treatments so they are discussed after 14 days of treatment. In addition, sparse collagen fibers were also observed in all treatments except negative control (Fig. 12d–f). On the other hand, after two weeks treatment, groups treated with Alg/CMC/ChNPs/DNPs (Fig. 12m–o) and Alg/CMC/ChNPs/DNPs/BTX-A (Fig. 12p–r) exhibited a densely epithelial thickness was appeared in Fig. 12 (arrow head). New blood vessels in Fig. 12 (thin red arrow) and hair follicle formation in Fig. 12 (thick black arrow) can be detected in these groups after two weeks of wound healing. Furthermore, bundles of collagen fibers (Fig. 12, star) in a random orientation were observed in Alg/CMC/ChNPs treated animals. Also, epidermal regeneration and new blood vessel and hair follicle generation can be observed in Fig. 12j–l. In treated animals with Alg/CMC/ChNPs hydrogel stretch and thicker bundles of collagen with a high population of cellular islands were observed in the reticular dermis in comparison with normal skin at the 14^th^ day which refers to a hypertrophic scar and was determined by the black star^37^. Sebaceous glands (Fig. 12, thick red arrow) and healthy epidermal layer are well-defined in normal skin (Fig. 12a–c). However, in negative control-treated animals, epidermal proliferation was not developed and the defect site was observed on the 14^th^ day. Hence, there was seen no other structure to compare but very few blood vessels. On the other hand, a crusty scab was seen in negative control and marked by a blue thick arrow in the negative control epidermal layer (Fig. 12d–f). Finally, in rat groups treated with Alg/CMC, the epidermal layer (black arrowhead) developed at a proper thickness with several blood vessels (thin red arrow) in the dermal layer Fig. 12g–i).

### Immunohistochemistry (IHC) staining

**Fig. 13:**
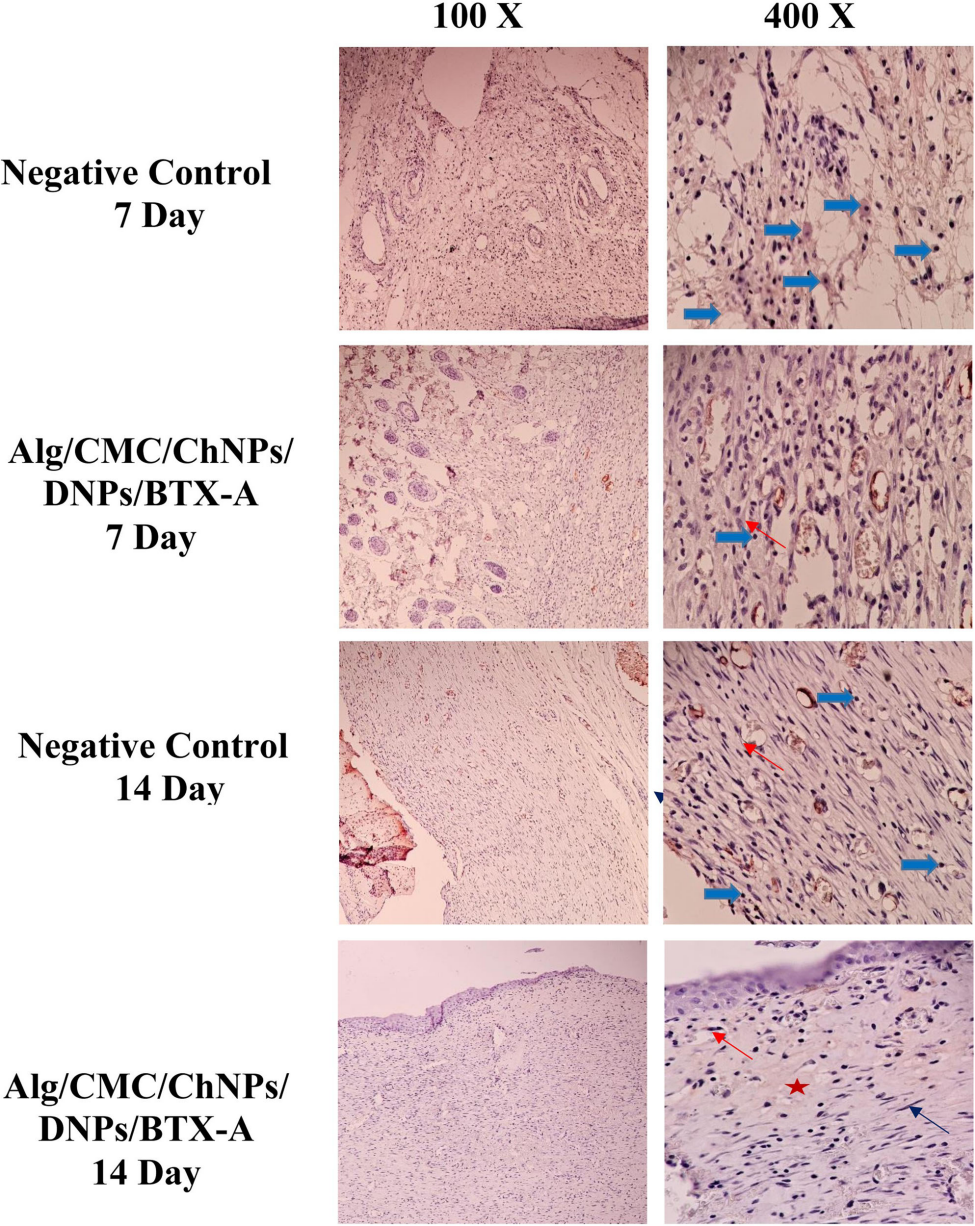
Immunostaining of TNF-α in the skin of rat treated with Alg/CMC/ChNPs/DNPs/BTX-A. Original magnification _100× & 400×. Thick blue arrow, thin red arrow, thin black arrow and red star indicated inflammatory cells, Fibroblast cells, fibrocyte and mature collagen respectively.

IHC of TNF-α was performed to assess the immunoreactivity of Alg/CMC/ChNPs/DNPs/BTX-A. TNF-α is responsible for regulating immune cell functions^38^. As shown in the (Fig. 13), the levels of TNF-α were highest on the 7^th^ day compared to the 14^th^ day for both the Alg/CMC/ChNPs/DNPs/BTX-A and negative control groups. Additionally, the negative control group exhibited higher levels of TNF-α and inflammatory cells compared to the Alg/CMC/ChNPs/DNPs/BTX-A group on the 7th day. Thick blue arrow, thin red arrow, thin black arrow and red star indicated inflammatory cells, Fibroblast cells, fibrocyte and mature collagen respectively.

**Fig. 14:**
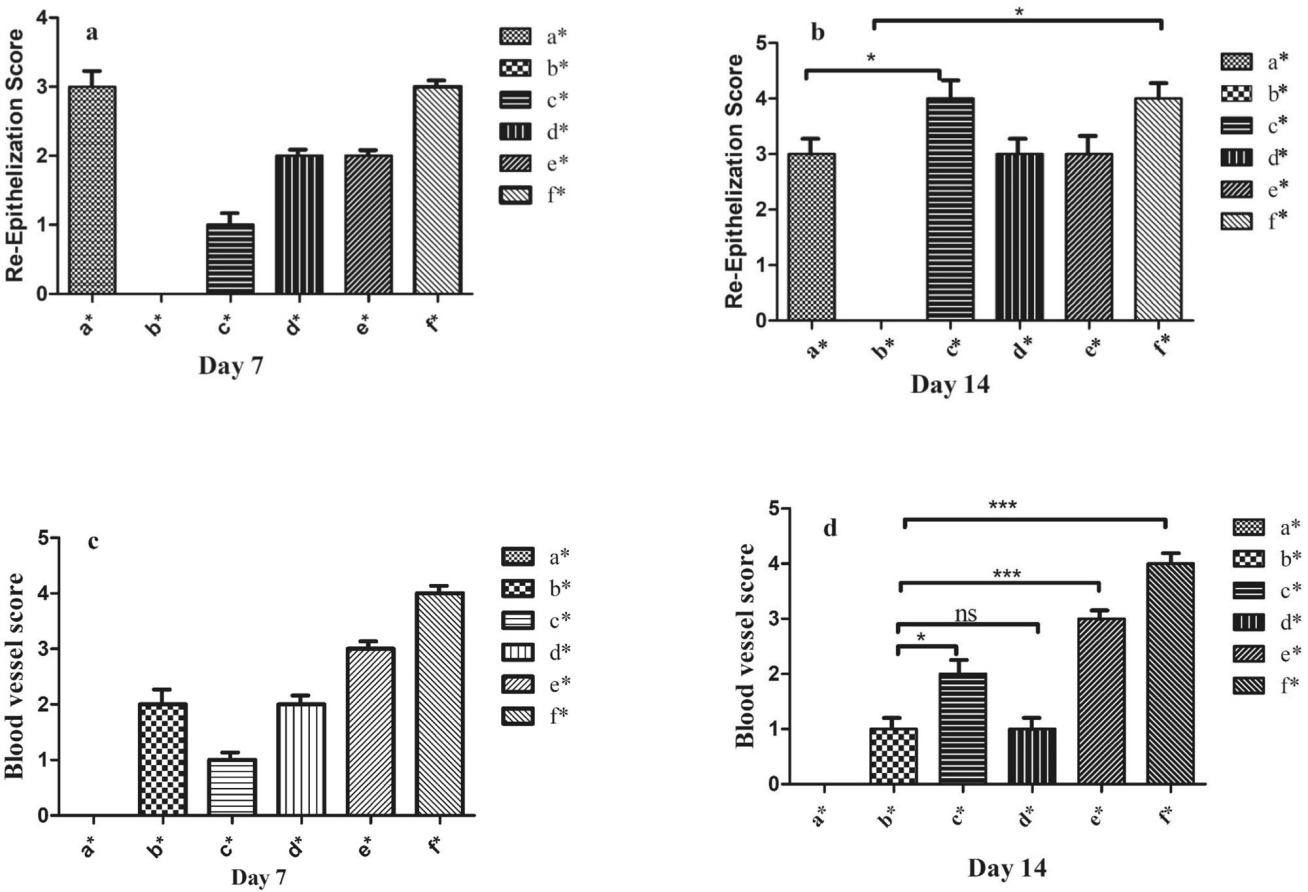
Re-epithelization diagram. Diagram study of re-epithelization (**a**, **b**) angiogenesis (**c**, **d**) of PC, positive control (a*) NC, negative control (b*), Alg/CMC (c*), Alg/CMC/ChNPs (d*), Alg/CMC/ChNPs/DNPs (e*), Alg/CMC/ChNPs/DNPs/BTX-A (f*). *P < 0.05 at 7^th^ day and 14^th^ day post treatment. Scores are identified as follows: 0 (without new epithelialization), 1 (25%), 2 (50%), 3 (75%), and 4 (100%).

Angiogenesis and re-epithelization analysis after one and two weeks post-injury has been illustrated in Fig. 14. Among all groups, on day 14 re-epithelialization in the negative control group (b*) was so close to the baseline and the Alg/CMC (c*) and Alg/CMC/ChNPs/DNPs/BTX-A (f*) obtained the greatest scores at about a similar degree (*P* < 0.05). While on day 7, among all groups Alg/CMC/ChNPs/DNPs/BTX-A (f*) indicated the highest score (Fig. 14a). Moreover, the most blood vessel score was assigned to Alg/CMC/ChNPs/DNPs/BTX-A (f*) groups in both one and two weeks after treatment (Fig. 14c, d). (*P* < 0.05).

### Quantitative real-time PCR

To study the skin wound healing process and scar improvements during healing after one- and two-weeks post-surgery, the gene expression levels of transforming growth factor β (TGF-β), insulin-like growth factor 1 (IGF-I), Smooth muscle alpha-actin (α-SMA, Act-A2), Versican (VCAN), Collagen Type I (COL1A1) was selected to be evaluated by RT-qPCR and data analysis was measured by Prism software version 5. GAPDH was used as a housekeeping gene in this study (Table 1). The results showed a correlation with histological assessments.

**Table 1:** Primers sequence

Genes	Primer sequence
rGAPDH-F	TCTCTGCTCCTCCCTGTTCTA
rGAPDH-R	ATGAAGGGGTCGTTGATGGC
rTGF-β1-F	AAGAAGTCACCCGCGTGCTA
rTGF-β1-R	TGTGTGATGTCTTTGGTTTTGTCA
rVCAN-F	CCCCTGCAACTACCACCTCACC
rVCAN-R	TCTTTCCAAAGGTCTTGGCATTTTCT
rIGF-1-F	GCTTTTACTTCAACAAGCCCACA
rIGF-1-R	TCAGCGGAGCACAGTACATC
rACTA2-F	AGCCAGTCGCCATCAGGAAC
rACTA2-R	GGGAGCATCATCACCAGCAA
rCOL1A1-F	CATGTTCAGCTTTGTGGACCT
rCOL1A1-R	GCAGCTGACTTCAGGGATGT

Primers used in qPCR.

*IGF* Insulin-like-growth factor, *TGF-β1* transforming growth factor beta 1, *VCAn Interleukin-6* Smooth muscle alpha-actin (α-SMA, Act-A2), *Col1A1* Collagen type I, *F* forward, *R* reverse.

### TGF-β1

**Fig. 15:**
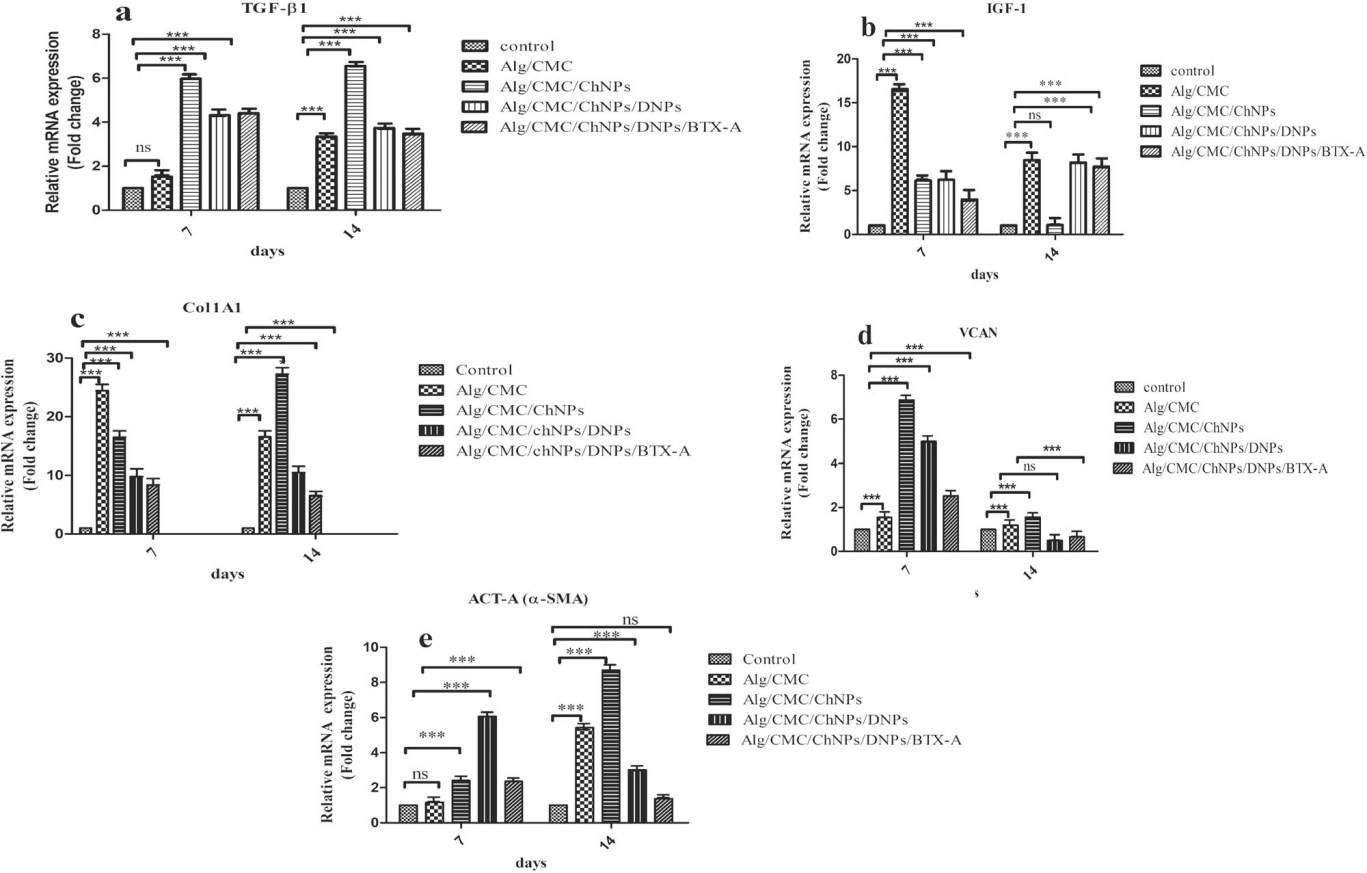
Gene expressions of TGF-βI, CoI1a1, IGF-I, Act-A (α-SMA), and VCAN (Versican) were analyzed by qRT-PCR. **a** TGF-βI, **b** IGF-I, **c** Col1a1, **d** VCAN, **e** Act-A expression in a rat model at 7^th^ and 14^th^ days after wound tissue treated with hydrogels and negative control. All groups were compared to the control group. Results are demonstrated as mean ± SD. (ns or no significant, *p < 0.05, ***p < 0.001).

The result of one-way ANOVA analysis exhibited a significant increase in TGF-β1 expression at the 7^th^ and 14^th^ days of healing relative to control in Alg/CMC and Alg/CMC containing ChNPs treatments groups. In comparison, TGF-β1 was significantly increased at 7^th^ days in all treatment groups relative to control. It also showed a drastic increase on day 14 in all groups relative to the control. Meanwhile, the level expression of TGF-β1 was a low upregulation compared to the 7^th^ day of wound healing in Alg/CMC/ChNPs/DNPs and Alg/CMC/ChNPs/DNPs /BTX-A. ***P < 0. 001 (Fig. 15a).

### IGF-I

Additionally, results of the IGF-I expression indicated a remarkable increase in all groups during one and two weeks of wound healing except Alg/CMC/ChNPs groups on 14 days. The expression level of the IGF-I on the 14^th^ day showed (a similar level) no difference relative to the control. *P* value = ns (no significant) and. ***P < 0.001 (Fig. 15b).

### Col1A1

The results demonstrated that Col1A1 gene expression was increased significantly in all groups of treatments relative to control on 7 and 14 days after treatments. Whereas, Alg/CMC/ChNPs/DNPs and Alg/CMC/ChNPs/DNPs/BTX-A showed less increase in Collagen type I expression on the 14^th^ day rather than that in Alg/CMC/ChNPs. ***P < 0.001 (Fig. 15c).

### Versican (VCAN)

Moreover, a drastic increase in the mRNA expression level of VCAN was observed in Alg/CMC/ChNPs and Alg/CMC/ChNPs/DNPs groups on day 7 relative to control, meanwhile, a one-and-a-half-fold increase in Alg/CMC and a 2-fold increase in Alg/CMC/DNPs/BTX-A treatments groups were reported at 7^th^ days relative to control. Also, a slight increase of versican was seen in Alg/CMC and Alg/CMC/ChNPs meanwhile almost a half reduction in the level of versican expression was reported in two other groups on day 14 relative to control may be related to the end of wound closure. Generally, the mRNA level of versican on the 14^th^ day was significantly low compared to the mRNA level of versican on the 7^th^ day (Fig. 15d). ***P < 0.001

### Acta-A (α-SMA)

In addition, our finding exhibited that on the 7^th^ day after treatments, a very low upregulation in Alg/CMC relative to control was observed. On the contrary, a 6-fold, 3-fold, and 3fold high expression of the alpha-smooth muscle actin (Act-A) was reported in Alg/CMC/ChNPs/DNPs, Alg/CMC/ChNPs, and Alg/CMC/ChNPs/DNPs/BTX-A relative to control, respectively (Fig. 15e).

A significant increase of Act-A level was seen in both Alg/CMC and Alg/CMC/ChNPs groups whereas a slight upregulation of Act-A expression was seen in Alg/CMC/ChNPs/DNPs, Alg/CMC/DNPs/BTX-A on day 14 of wound healing. In this regard, Alg/CMC/ChNPs/DNPs, Alg/CMC/ChNPs/DNPs/BTX-A showed lower Act-A level expression on 14^th^ day compared to the 7^th^ day after treatments). ***P < 0.001 and ns non-significant.

## Discussion

In recent years, skin tissue engineering has been extensively developed by designing potential and effective wound healing products to promote the remodeling of skin tissue. Furthermore, hydrogel-based wound dressings are highly considered an appropriate material in favor of wound closure, angiogenesis, and scar-free are also importantly required for tissue regenerative^39^. Successful tissue regeneration can be achieved by materials such as bioactive glass nanoparticles, which provide a similar membrane to desired tissue in beneficial cell proliferation and migration^40^. More importantly, the placenta membrane has low antigenic, highly anti-inflammatory, and representing anti-scar properties. These features are remarkably beneficial for functional tissue restoration^41^. According to the anti-scar properties of botulinum toxin A, it is extensively used in therapeutic approaches as an effective agent for hastening scar-free wound repair^42^. The study aimed to create a multifunctional wound dressing using Alg/CMC hydrogel with ChNPs, DNP, and BTX-A to speed up wound closure, promote blood vessel growth, and reduce scarring in Wistar rats. ChNPs (10%w/w) were combined with Alg/CMC (2:1), along with 200 µl/10 ml BTX-A and 0.1%w/v DNPs, and crosslinked with CaCl2 (75 mM). The study includes discussions on nanoparticle characterization, decellularization assay quantification, in vitro, and in vivo assessments.

In the present study, the results of the H and E and DAPI staining of HCM and dHCM indicated that dHCM was successful. SEM investigation of dHCM structure corroborated the results obtained from DNA content assay and DAPI staining. Although, it is claimed that a DNA content percentage value from 0.2 to 0.4%, exhibited no likelihood of transplant rejection and considered is an ideal range, however greater residual DNA percentage of up to 10% is also reported as acceptable for the stable recipient^43,44^. Similar to the present study, residual DNA percent is reported 3% which is a desirable percent to use in clinical applications.

The average hydrodynamic size and mean value distribution of nanoparticles are determined by DLS. Zeta potential is mainly related to the surface ions of nanoparticles and the zeta value between −30 to +30 mv is an acceptable range for confirming the stability of particles. Besides Zeta potential of nanoparticles from −10 to + 10 is considered neutral and strongly stable^45^. Although it is stated that nanoparticles from 0 to 100 nm represent narrow distribution in size with specific characteristics, this value is defined differently related to various material. Nanostructures greater than 100 nm are also discovered to have very unique and non-toxic properties according to literature similar to the present study^46^.

Moreover, PDI below the value of 0.5 exhibits an excellent monodispersity and homogeneity of synthesized nanoparticles, similar to PDI of DNPs and ChNPs in this study^47^. Similar to this study, PDI values of 0.5 and 0.4 in ChNPs and DNPs also confirmed that nanoparticles were ideally synthesized monodispersed.

In FTIR test the broad absorption peak of DNPs hydrogel at about 3300 cm^−1^ to Alg/CMC alone exhibited the interaction between the cation in DNPs structure and O-H groups of Alginate and CMC hydrogel besides two peaks at 2000 ascribed to C-H was disappeared can be shown that strong hydrogen bonds emerged after DNPs was added to the hydrogel. In addition, the distinctive peaks of C-O-O were shifted to the lower wavenumbers indicating the interaction of cation and carboxylate groups^31^. Furthermore, methylene groups are specific to the structure of BTX-A, located between lysine and cysteine^32^. On the other hand, there are coordinated bending modes between the functional groups of C-O-O stretching vibration in Alg/CMC/BTX-A and pure Alg/CMC, resulting in a small shift to a weaker and lower absorption peak. However, the relative intensity closely overlapped, making it difficult to discuss^48^. The results demonstrated that two peaks around 1600 and 1400 cm^−1^ shifted to higher wavenumber and three peaks at 2800, 1500, and 600 cm^−1^ disappeared in Alg/CMC/ChNPS/DNP/BTX-A indicating the presence of strong interaction between ions and functional groups of Alg/CMC with diopside nanoparticles, BTX-A, and chorion nanoparticles. Besides a slight shift in peak ranges related to a slight overlapped of vibration bands in the combination structure. Also, the presence of Mg-O peak in Alg/CMC/ChNPS/DNP/BTX-A confirms the successful blending of diopside nanoparticles with BTX-A, ChNPs, and Alg/CMC hydrogel.

As previous studies described^49–51^, hydrophilic groups in hydrogel structure are responsible for water uptake capacity. A higher water absorption facilitates cell proliferation and has a positive impact on nutrient transmission to the wound site^52^. Therefore, we hypothesized that the molecular interaction in the structure of Alg/CMC/DNPs was stronger due to divalent cations such as Mg^2+^ and Ca^2+^with functional groups of hydrogels and indicating more resistance to water absorption compared to Alg/CMC hydrogel. In addition, egg box structure conformation is attributed to interactions between cations and G residue in the network of Alg/CMC hydrogel through hydrogen bonds, it causes restriction in intermolecular interaction. Although a previous study^53^ claimed that dHCM showed a high water uptake capacity, in this research Alg/CMC hydrogel containing ChNPs in combination with DNPs and BTX-A showed a gradual increase until the first days after immersions may be attributed to the restriction of functional groups of DNPs ions and BTX-A interacted with functional groups of chorion nanoparticles. Meanwhile, it is believed that a rapid increase in water uptake after 24 h may be indicated that DNPs ions were released to PBS gradually, and changes in BTX-A functional group activity would be responsible for the high-water absorption. Finally, it is concluded that Alg/CMC/ChNPs/DNPs/BTX-A is highly stable for 2 days and it is suitable for cell adhesion.

On the contrary, the water uptake property of BTX-A has not been investigated and is not clearly understood. Basically, at initial hours BTX-A hydrogel absorbed a high content of water at once, however, an abrupt decrease occurred after 48 h of immersions in PBS due to polymer hydrolysis reaction. Notably, to further discover the further mechanism of BTX-A interaction more investigations are required.

The degradation rate of hydrogel is significantly important for a successful healing rate of a damaged tissue in a favorable time manner^50^. Based on a previous study^54^ hydrogel containing DNPs exhibited a lower degradation rate than that of BTX-A hydrogel and Alg/CMC alone due to the strong linkage between ions and functional groups of DNPs and Alg/CMC hydrogel. On the other hand, by adding BTX-A to Alg/CMC hydrogel a strong repulsive force emerged between the network chains of polymers. In brief, due to the literature^55^, crosslinking density changed the degradation rate of the structure so that the poor stability between hydrogel functional groups chains led to a high degradation rate of BTX-A hydrogel after 2 days, and no further measurable weight ratio was observed after 3 days. It is believed that strong bonds in Alg/CMC/ChNPs/DNPs/BTX-A structure contribute to the slow degradation rate of this hydrogel and suddenly after 48 h, the ions are released into PBS. Additionally, the inclusion of BTX-A and ChNP nanoparticles in the hydrogel resulted in their placement between the interacting molecular chains of Alg-CMC, significantly impacting the structure by enhancing the interconnection of hydrogel networks. This led to a weakened intermolecular connection between the hydrogel chains, causing a high degradation rate. After 48 h, there was no discernible hydrogel remaining. When the numbers were input into the formula, there was no “W1” to replace in the formula.

Interestingly, in the present study with a comparison of the blood compatibility effect between BTX-A hydrogels and Alg/CMC, the results displayed a higher blood coagulation index percent rather than that of Alg /CMC alone. A case report study declared that an overdose injection of BTX-A lead to a high level of prothrombin time by coagulation assay according to clinical reports^56^. Furthermore, BC investigation of Alg/CMC/ChNPs/DNPs/BTX-A was 5.8% representing excellent blood compatibility of this sample, however, a small increase in BCI percentage may be related to the low blood clotting property of chorion nanoparticles. In a previous study, dHCM was evaluated as a proangiogenic factor and the hemolysis rate of the trophoblast layer side was reported 5.02 ± 1.26% so similar to our results^57^. More importantly, it should be considered that Alg/CMC/DNP/CNP-BTX-A was evaluated, in this study.

Although the blood compatibility test of 200 µl BTX-A /10 ml hydrogel was 6.8%, it is considered an acceptable value relatively close to the Alg/CMC, as the standard percent for BC tests. Blood compatibility assay refers to the effect of treated hydrogels on erythrocytes and can support wound closure due to hemostasis property which is identified as less than 5%^58^. According to previous research^59^, the results obtained from BC and BCI investigation of DNPs/Alg/CMC compared to Alg/CMC confirmed that DNPs displayed an excellent blood compatibility effect.

It is well-documented that protein denaturation is associated with the inflammation process. The primary mechanism of NSAID action has been attributed to the inhibition of protein denaturation^60^. According to the literature^61^, Placenta extract offers protection and is linked to reduced serum IgE levels and inflammation. Johnson et al. ^62^ demonstrated that viable cryopreserved human amniotic membrane (vCHAM) and dHACM displayed anti-inflammatory activity. Additionally, our findings revealed that chorion nanoparticles effectively inhibited thermally induced albumin denaturation at all tested concentrations, indicating their potential to control protein denaturation linked to inflammation. However, further investigation is necessary as there were no similar tests of protein denaturation and studies on the anti-inflammatory potential of chorion. Although aspirin demonstrated higher concentration, ChNPs at higher concentrations showed a proper inhibitory effect of 77%. However, the presence of more than 50% inhibitory activity confirmed the anti-inflammatory ability due to the multiple components in the structure.

Prior studies have shown that bioglass-based structures not only have the ability to form tube networks in in-vitro assays of HUVEC cells but also increase the number of tube-like structures compared to the control group^59,63^. Similarly, our finding indicated the tube-like structure formation capacity of 0.1%w/v DNPs. This may be due to the release of cation, such as Ca^2+^, Mg^2+^, and Si^4+^ from the diopside, creating a suitable environment for accelerating angiogenesis activity and contributing to the improvement of wound healing by providing sufficient nutrients and oxygen. While Xie et al. ^64^ found a low level of proangiogenic activity in Cerium-Containing Bioactive Glasses, our research observed a high number of tube-like structures after 12 h of treatment.

Cytotoxicity is a crucial factor in wound dressing fabrication. More importantly, it can support skin tissue restoration^65^. To evaluate the cell viability of ChNPs, DNPs, and BTX-A hydrogel in comparison with pure Alg/CMC, the MTT test was conducted. In this study, the results confirmed that all samples not only increased cell proliferation percent after 72 h but also exhibited no cell toxicity on 1^st^ and 3^rd^ days post treatments.

Despite of inhibition effect of BTX-A fibroblast cells derived hypertrophic scars^66^, at 200 µl/10 ml concentration in this study, it showed a higher proliferation rate on 3T3 cell lines than DNPs hydrogels. It is conceived the botulinum toxin A represented a dose manner effect.

In a previous study, IHC staining of full-thickness cutaneous wound proved that treated groups at day 14 showed lower TNF-α rather than treated groups at the 7^th^ day according to the inflammatory phase of the wound healing process^38^. In another study, to further validate inflammatory factor in early phases on day 3, the phenotype of TNF-α was measured using immunohistochemistry assay. While the level of TNF-α on day 3 was substantially higher in the curcumin-treated wounds than in the control groups. On the contrary, a low level of TNF-α was observed on days 7 and 12 compared with those in the control group^67^. Similar to the literature our results indicate that the Alg/CMC/ChNPs/DNPs/BTX-A hydrogel showed an anti-inflammatory effect and showed no immunoreactivity. Thus, the Alg/CMC/ChNPs/DNPs/BTX-A hydrogel group could inhibit inflammatory reactions in the wound healing process. These results were associated with H and E staining results on the 7^th^ and 14^th^ day.

In general, in vivo studies showed that the healing ability and epithelial thickness in Alg/CMC/ChNPs/DNPs/BTX-A hydrogel were more acceptable and resemblance to normal tissue than in the other groups. Furthermore, blood vessel formation in Alg/CMC/ChNPs/DNPs/BTX-A and Alg/CMC/DNPs rat treatment groups was surprisingly increased. Thus, these groups provide a sufficient condition for further remodeling, and healing tissues in these groups were more resemblance to normal skin.

The new vascular network plays a crucial role in the cutaneous wound healing process in supply nutrients and oxygen at the wound bed, and subsequently, contributing to a new regeneration of granulation tissue^34^. In our work, neovascularization and re-epithelialization of Alg/CMC/ChNPs/DNPs/BTX-A were detected as more effective for tissue regeneration. Besides, the wound closure rate was faster in Alg/CMC/ChNPs/DNPs and Alg/CMC/ChNPs/DNPs/BTX-A groups. In the current study, the margin of wound area in Alg/CMC/ChNPs/DNPs/BTX-A almost disappeared on day 14. Therefore, this group is suggested as the most favorable treatment in full-thickness wound healing.

To further study of skin wound healing process and scar improvement during healing after one- and two-weeks post-surgery, the gene expression levels of transforming growth factor β (TGF-β), insulin-like growth factor 1 (IGF-1), α- smooth muscle actin (α –SMA or Act-A2), Versican (VCAN), Collagen Type I (COL1A1) was selected to be evaluated by RT-qPCR.

It is stated that IGF-1 and TGF-β1 promote angiogenesis and control collagen deposition during the wound healing process. They played a major role in regulating tissue repair progress^68,69^. It is claimed that prolonged upregulation of TGF-β1 after 7^th^ days contributes to hypertrophic scar formation, hence the expression level of TGF-β1 is time-dependent and indicated the wound age^70–72^. Also, a previous study displayed that TGF-β1 expression levels decreased relative to that of normal skin at 14^th^ days after wound treatment, however, a high level of TGF-β1 was observed at 5th days post-wound injury^73^. This result is similar to the expression level of TGF-β1 in our study. We demonstrated that in treated rats with Alg/CMC containing ChNPs, DNPs, and BTX-A, TGF-β1 expression highly increased on the 7^th^ day. Afterward, a low level of TGF-β1 expressions was seen on day 14, representing efficiently wound healing.

Interestingly, IGF1 is responsible for cell survival, proliferation, differentiation, and modulating keratinocyte and fibroblast proliferation, and plays main roles in angiogenesis, and regulation of collagen synthesis^74^. As described in a previous study, it is demonstrated that IGF-I level was moderately increased at the latest stage of the wound healing process^75^. In the present study, in all treated groups we showed that IGF expression was increased during wound healing and assisted to accelerate wound closure and tissue repair. Collagen type I is a prominent component of ECM involved in the initial stages of the wound healing process and maintains skin integrity. Based on a further study, Col1A1 was highly expressed on day 8 post-treatment, whereas exhibited a decrease after 12^th^ days of treatment. In this study, a reduction in area wounds was obvious on day 12^76^. Despite Versican expression being upregulated at the early steps of wound healing, remaining at a high level after the inflammatory phase of the wound healing process represented pathological disorder and delay in the healing process on time. According to a previous study, it is expected to be expressed instantly 2 days post-injury and reached the highest level on day 7, and keep increased trend by day 14, a declining level should be observed thereafter. Overexpressed VCAN after day 14 contributes to hypertrophic scar or keloid formation resulting in increasing smooth muscle α-actin gene expressions which are defined as the specific highlight marker for myofibroblast marker^77^. These findings correlated with our study, versican showed a high level of mRNA expression relative to control on the 7^th^ day. It showed a very low increase on day 14 relative to control and become so close to the baseline of control.

Accordingly, to the literature, smooth muscle α-actin (Act-A2) is well known as a highlight marker of myofibroblast which is expressed at the wound site, indicating wound contraction with increasing collagen deposition. It also remains expressed at a high level in keloid scars even after the end of wound closure. In another study, it is demonstrated that fold change of α- SMA was reported at a high level on the 7^th^ day post-injury. However, on the 14^th^ day post-wounding when granulation tissue was generated α- SMA level reduced, and on the 21^st^ was disappeared^78^. In our study, regarding the anti-scarring properties of Alg/CMC/ChNP/DNPs/BTX-A, the wound healing process improved faster, besides a high reduction in α- SMA expression at 2 weeks after treatments were observed.

Taken together, in our study in treated groups with Alg/CMC/ChNPs/DNPs/BTX-A Col1A1expression level on day 14 was decreased. In this group, the wound healing process and wound closure rate showed an extensive increase compared to the others. All real-time PCR data were correlated with histological assessments.

In this study, Chorion membrane, ChNPs, and DNPs nanoparticles were prepared after decellularization. Subsequently, DNPs and BTX-A-based hydrogel were prepared, and in-vitro evaluations were conducted. The results of the cell-viability study showed no toxicity in fibroblast cells treated with Alg/CMC groups containing DNPs and BTX-A. In in-vivo experiments, Alg/CMC/ChNPs/DNPs/BTX-A hydrogel demonstrated better regeneration of granulation tissue compared to other groups. Additionally, Alg/CMC/ChNPs/DNPs/BTX-A exhibited the highest re-epithelization and neovascularization scores. Gene expression analysis confirmed these histological results. TGF-β1 showed a significant increase on day 7 and a rapid reduction on day 14 in Alg/CMC/ChNPs/DNPs/BTX-A compared to controls. Generally, Alg/CMC/ChNPs/DNPs and Alg/CMC/ChNPs/DNPs/BTX-A showed similar mRNA expression results for Versican, IGF-I, and TGF-β1, except for Col1A1 and Acta-A expressions. Despite the low expression of Acta-A (alfa -SMA), our findings indicated that the Alg/CMC/ChNPs/DNPs/BTX-A group had a higher wound closure rate than the other groups. In summary, this study demonstrated the potential of Alg/CMC hydrogel containing ChNPs, DNPs, and BTX-A in regenerating cutaneous wound healing. It can be considered as a feasible and effective therapeutic approach.

## Methods

### Human chorion membrane

Following written informed consent, human placenta samples were collected from the elective cesarean section in Bahar Hospital according to the ethical committee protocol of Shahroud University of medical sciences (IR.SHMU.REC.1400.179). All donors were serologically negative for human immunodeficiency virus (HIV), and hepatitis virus type B. The placenta was stored in phosphate-buffered saline (PBS, pH 7.4) containing 1% streptomycin/amphotericin B and washed several times using this solution. Finally, the chorion membrane was separated from the amnion membrane. All process steps were conducted in sterile conditions.

### Decellularization of human chorion membrane

Regarding a previous study on the human chorion membrane decellularization process described by Frazão^53^. The HCM was peeled off and cut into smaller pieces, the samples were washed several times with PBS to eliminate any blood. Then, the membranes were subjected to two freezing/thawing cycles changing from −80 to 37 °C respectively. Afterward, the pieces were submerged three times into 0.5% SDS for 3 h each. After that, the membranes were treated overnight with 0.1% SDS. Then the HCM pieces were incubated with 1% Triton-X100 three times at ambient temperature for 30 min each, followed by gentle scraping on both sides to remove the trophoblast layer completely. Three washes of 15 min were performed with 0.9% normal saline. Next, the samples were treated with DNase I (50 U/CC) for 30 min at 37 °C, followed by 30 min treated with 0.1% SDS at 4 °C. After three washes with PBS, decellularized HCM were stored at −20 °C for further experiments.

### Preparation of chorion nanoparticles

To prepare chorion nanoparticles, the freeze-dried decellularized membranes were milled for about 3 h and 20 min at 300 rpm.

### Preparation of diopside nanoparticles

Diopside nanoparticles were synthesized according to the sol-gel method^79^. Briefly, a mixture of magnesium chloride hexahydrate (MgCl2·6H2O), calcium nitrate tetrahydrate (Ca (NO3)2·4H2O) at the molar ratio of 1:1 were slowly dissolved in 150 ml absolute ethanol and vigorously stirred for 30 min at 70 °C to obtain a homogenous solution. Then tetraethyl orthosilicate (TEOS) 0.25 mole was blended with the solution and stirred for 2 h at 400 rpm. pH of diopside nanoparticles was adjusted to 8 by adding dropwise of NH3 (25%) to the solution and stirring gently for 1 h. The wet gel emerged at the final of this step. Finally, the wet gel was dried at 70 °C for 24 h and sintered at 500 °C for 2 h. Subsequently, the weighed powders were milled in a planetary ball mill for 4 h and 20 min at the rotation speed of 300 rpm.

### Preparation of crosslinked Alg-CMC hydrogel containing diopside nanoparticles/chorion nanoparticles/ BTX-A

Sodium alginate and CMC (3%w/v) with a ratio of 2/1 were dissolved in distilled water and mixed under magnetically stirring for 24 h. Then, 200 µl, 0.1%w/v, 10% w/v of BTX-A, diopside nanoparticles DNP, and chorion nanoparticles (ChNP) were added into the 10 ml Alg/CMC solutions, respectively, and stirred overnight at 400 rpm. Afterward, calcium chlorides (CaCl2) 75 mM was used to crosslink the hydrogel samples.

### Characterization of the scaffolds

#### Morphology study

The morphology of the chorion and diopside nanoparticles was studied using scanning electron microscopy (SERON TECHNOLOGY, AIS2100, South korea) with a voltage of 20 KV and various magnifications. The samples were prepared with gold metal coating under a high vacuum for 250 s using a sputter coater.

#### DNA extraction and quantification

Cell and Tissue DNA isolation kit (FPKT023.0050, Kiagene Fanavar Aria co,Iran) was used to extract total DNA. Both native and decellularized membranes were crushed using liquid nitrogen and weighed according to the manufacturer’s instructions. The quantification of DNA was carried out using the (BioTek Cytation multimode reader, USA) according to the manufacturer’s instructions.

#### DAPI staining

In the present study, 4′, 6-diamidino-2-phenylindole (DAPI) staining was used for the evaluation of nucleus properties and the absence of cellular structures confirmation in the decellularized chorion membrane. The micrograph was captured by an inverted fluorescent microscope (Olympus IX71 and fluorescence-RFL-T, Tokyo, Japan).

#### Characterization size distribution and zeta potential of nanoparticles

The average hydrodynamic diameters (HD), polydispersity index (PDI), and Zeta potentials of the chorion and diopside nanoparticles were determined after re-dispersion of samples in distilled water by Malvern Zeta Sizer Nano ZS (Malvern Instruments Ltd., UK) in triplicate.

#### FTIR (Fourier-transformed infrared spectroscopy) test

FTIR was performed to characterize the reaction between functional groups in the hydrogel structures containing DNPs and BTX-A in comparison with Alg/CMC alone. FTIR spectra were recorded within the range between 300 cm^−1^ and 4000 cm^−1^, using an ATR-FTIR spectroscopy method (Spectrum GX, PerkinElmer, USA).

#### Water uptake capacity (WUC)

The WUC of hydrogel samples was measured in the PBS solution (pH = 7.4), at RT, representing the capacity of fabricated hydrogel to absorb wound exudates. The lyophilized hydrogels were prepared using a freeze drier. Initially, the freeze-dried samples were weighed (W0) and immersed in the specific amount of PBS and kept at ambient temperature for various time points (1, 3, 6, 12, 24, 48, 72 h). Subsequently, at determined time points the hydrogel samples were weighed (W1) and then the results were measured based on (Eq. (1)).

1 $${\rm{Water}}\,{\rm{Uptake}}={\rm{W}}1-{\rm{W}}0/{\rm{W}}0\times 100$$

Where W0 and W1 are the dried mass weight and the weight of hydrogels after absorbing the solution, respectively.

#### Weight loss analysis

The degradation rate of hydrogel samples was measured by weight loss tests. Freeze-dried hydrogels were preliminarily weighed (W0). Then, all samples were immersed in PBS solution (pH = 7.4). After 24, 48, and 72 h, the excess solution was removed and dried hydrogel samples were weighed (W1). Then the degree of weight loss was measured based on (Eq. (2))

2 $${\rm{Weight}}\,{\rm{loss}}\, \% =\frac{W0-W1}{{\rm{W}}0}\times 100$$

#### Blood compatibility and blood coagulation index

According to previous reports^34,80^, a blood compatibility test was performed to investigate the blood compatibility of the samples. Adult volunteer’s informed consent to collect their blood samples. Initially, 2 ml of the collected blood was diluted by 2.5 ml of normal saline and gently mixed. After that 4 ml distilled water blended with 200 µl of diluted blood and 4 ml normal saline added to 200 µl of diluted blood are considered as positive control and negative control respectively. Next, 100 µl of each hydrogel sample was placed into 96 well plates in triplicate. 200 µl of prepared mixed blood was added to each well. After that the plates were placed into a 37 °C incubator for 1 h. Finally centrifuged at 1500 rpm for 10 min. After transferring the supernatant to another well, the absorbance was measured at 545 nm.

The percentage of hemolysis was calculated by the following (Eq. (3)).

3 $${\rm{Blood}}\,{\rm{blood}}\,{\rm{compatibility}}\,{\rm{or}}\,{\rm{hemolysis}}\,( \% )=({\rm{ODs}}-{\rm{ODn}})/{\rm{ODp}}-{\rm{ODn}}\times 100$$

Where ODs is the absorbance of a tested sample, ODn refers to the absorbance of the negative control, and ODp is the absorbance of the positive control.

To determine the blood clotting index of hydrogel samples, diluting fresh human blood in the citrated tube was prepared as described in previously published protocols^81^. At first, 1.5 ml of each sample were incubated in 25 ml beakers and placed into a water bath at 37 °C for 1 h. Afterward, the samples were carefully incubated with 100 µl of fresh human blood. After 5 min 0.2 mol/l, CaCl2 was added into each beaker. Then, 25 ml of distilled water was slowly poured onto the sample after 5 min. Ultimately, the sample was incubated for 5 min, and suspensions were transferred into a 96-well plate.

The control was prepared by adding 100 µl of fresh human in 25 ml deionized water.

The blood clotting index was evaluated by spectrophotometric measurement of the relative absorbance of hydrogel samples at 545 nm.

The blood clotting index (BCI) value of hydrogel samples was quantified by (Eq. (4)):

4 $${\rm{Blood}}\,{\rm{clotting}}\,{\rm{index}}\,({\rm{BCI}} \% )\!:{\rm{Abs}}\,{\rm{sample}}/{\rm{Abs}}\,{\rm{control}}\times 100$$

Where Abs sample and Abs control are the absorbance of treated samples and the released hemoglobin of erythrocytes after contact with the diluted water, respectively.

### In-vitro anti-inflammatory activity

#### Inhibition of protein denaturation

The potential for inhibiting albumin denaturation was assessed according to the literature^82–84^. A reaction mixture (5 mL) containing 0.2 mL of BSA (Bovine serum albumin), 2.8 mL of PBS (pH 6.4), and 2 mL of determined concentrations of ChNPs (10, 50, 100, 500, 1000 mg/10 mL) were incubated for 15 min at 37 °C, then heated up to 70 °C for 5 min. Distilled water at a similar volume was prepared as a control. Finally, the absorbance of samples was read at 660 nm. Acetylsalicylic acid was used as a reference drug at concentrations of 5, 10, 15, 25, 50, and 100 mg/mL, treated similarly and measured at the same absorbance. The percentage inhibition of protein denaturation was calculated using the following (Eq. (5)):

5 $${\rm{Inhibition}}\,{\rm{of}}\,{\rm{protein}}\,{\rm{denaturation}}\,( \% )={\rm{A}}\,{\rm{Control}}\_{\rm{A}}\,{\rm{test}}/{\rm{A}}\,{\rm{control}}\times 100 \%$$

Where A control = absorption of the control sample, and A test = absorption of the test sample.

#### HUVEC tube formation assay

For the in vitro study of angiogenesis according to the literatures^59,85^, 2 × 10^4^ HUVECs cells/ml were seeded for 24 hours into each well of a 24-well plate previously coated with 100 μL of Matrigel for 1 h at 37 °C in a 5% CO2 incubator to allow cell attachment. After 24 h, the cells’ culture medium was replaced by the test media containing 0.1% w/v DNPs, and tube formation was examined after 12 h of incubation and photographed using an inverted microscope (Nikon, Tokyo, Japan) at 10× magnification.

#### Cell viability

The indirect MTT assay was performed to study the cytotoxicity of hydrogel samples. Fibroblast cell line (3T3 cell line) was achieved from the Pasteur Institute’s National Cell Bank of Iran. In brief, to perform indirect MTT, sample extractions were first exposed to DMEM at 24, 48, and 72 h in sterile conditions, separately. After that, the 3T3 cell lines were cultured in a sterile 96-well plate containing DMEM media with 10% fetal bovine serum at a density of 1 × 10^4^ cells on each well at 37 °C in a 5% CO2 incubator. After overnight incubation, 120 µl extract of crosslinked alginate/CMC/ChNPs/DNPs with and without BTX-A at various concentrations were added separately to each well, in triplicate, and incubated for 24 hours in a 5% CO2 incubator at 37 °C. After that, supernatants were removed and 20 µl MTT solution (5 mg/ml) was added to each well. After 3 h incubation at 37 °C, DMSO solution (150 µl) was added to dissolve the formazan crystals. The absorbance was recorded by a plate reader at 570 nm. The cell viability percentage of prepared samples was calculated by the following (Eq. (6)):

6 $${\rm{Cell}}\,{\rm{viability}}\,( \% )={\rm{Abs}}\,{\rm{sample}}/{\rm{Abs}}\,{\rm{control}}\times 100$$

#### In vivo experiment

24 adults male Wistar rats weighing (200–220 g) were purchased from Shahroud university medical sciences and housed under standard conditions, Food and water were available ad libitum and animals were maintained at a controlled room temperature (22.0 ± 0.5 °C) at 12/12 h light/dark cycle in individual cages. All procedures were approved by the ethical guidelines committee at Shahroud medical university (IR.SHMU.REC.1400.179).

#### In vivo wound model

Animals were anesthetized by 100 mg/kg ketamine/10 mg/kg xylazine intraperitoneal injection for each animal. The rats were shaved at the back cleaned and sterilized with Betadine®. The wound area was marked and a 1.50 × 1.50 cm^2^ full-thickness skin excision was created using a scalpel blade.

Animals were randomly divided into six groups containing four animals in each group and housed individually after surgery. Group, I: Positive control (without surgery and treatment), Group II: was treated with Alg/CMC hydrogel, Group III: received Alg/CMC/ChNPs (10% w/w), Group IV: treated with Alg/CMC/10%W/W ChNPs/0.1% w/v DNPs hydrogel, group V: treated with Alg/CMC/ChNPs/DNPs/10 µl BTX-A hydrogel, and group VI: negative control (received no treatments but sterile gauze). The wounds were covered with 500 µl of prepared hydrogels in groups II–V for each animal. Treatments were performed every other day. After 7- and 14-days post-surgery, animals were euthanized by overdosed ketamine (200 mg/kg) injection, and tissue sections surrounding healing were explanted for subsequent histological examination. Wound contraction was investigated as a percentage reduction of wound bed size.

#### Histology, immunochemistry and image analysis

The structure characteristics of the acellular chorion membrane were stained and checked by hematoxylin and eosin (HE). All treated and untreated rat groups were euthanized by ketamine 200 mg/kg intraperitoneal injection after 7 and 14 d post-surgery. 10% formalin solution was used to fix the harvested tissue specimens for histopathological assessments. All samples, were gradually dehydrated, embedded in paraffin, and sliced into 5 μm thickness sections. Then all tissues were stained by both hematoxylin and eosin for histological examination.

H&E staining was used to assess epithelialization, collagen deposition, scar formation, angiogenesis, and granulation tissue formation evaluated in different groups, comparatively. Finally, the histological slides were observed under a light microscope (Olympus BX51; Olympus, Tokyo, Japan).

The immunohistochemistry of skin sample sections was performed as described in the previous methods^67,86^ to assess the immunoreactivity of Alg/CMC/ChNPs/DNPs/BTX-A compared to the control. The samples were treated with rabbit monoclonal anti-mouse TNF-α antibody (1:200 dilution; Abcam, Cambridge, Massachusetts) overnight at 4 °C. Then, the sections were washed with PBS and incubated for 1 h at room temperature with biotin-conjugated goat anti-rabbit IgG (1:200; Vector Laboratories, Peterborough, UK). Subsequently, the samples were imaged using light microscopy (Olympus BX51; Olympus, Tokyo, Japan).

#### Wound closure and re-epithelization percentage

Further, the wound closure percentage and re-epithelization rate were determined as described in previous studies^80^. Re-epithelialization and wound closure were monitored photographically at the 1, 3, 10-, and 14 days post-surgery. Ultimately, the results were expressed by ImageJ software (NIH, USA). The wound closure and re-epithelization percentage were calculated via Eqs. (7) and (8) respectively. Re-epithelialization measurement and new blood vessel formation are the major for comparing and evaluating the effectiveness of treatments on tissue regeneration. In this study, to evaluate re-epithelialization value on day 7 and 14, a 5-point scale was determined as a semi-quantitative assay: 0 (without new epithelialization), 1 (25%), 2 (50%), 3 (75%), and 4 (100%). New blood vessel scales are characterized similarly to re-epithelialization scores

7 $${\rm{Wound}}\,{\rm{closure}}\,( \% )=\left(1-\frac{{open\; wound\; area}}{{initial\; wound\; area}}\right)\times 100$$

8 $${\rm{Wound}}\,\mathrm{Re}{\hbox{-}}{\rm{epithelization}}\,( \% )={\rm{distance}}\,{\rm{re}}{\hbox{-}}{\rm{epithelization}}/{\rm{wound}}\,{\rm{area}}\times 100$$

#### RNA extraction and quantitative real-time polymerase chain reaction (PCR)

Total RNA was extracted (RNX-Plus Solution Kit, Iran) from wound explanted tissues. After cDNA synthesized by Easy cDNA Synthesis Kit cDNA Synthesis Kit ;) Parstous, Iran), reverse transcription quantitative RT-PCR was carried out using SYBR® Green Real Time PCR Master Mix (Amplicon). The expression levels of TGF beta1, IGF, VCAN, ACTA (alfa-SMA), COL1, and GAPDH genes in treated and control groups were detected using a quantitative polymerase chain reaction (Q-PCR). GAPDH was used as a housekeeping gene. The primers used in this study are summarized in Table 1: Relative quantity of mRNA expression was calculated by 2^−ΔΔCt.^ All RT-qPCR experiments were evaluated using the Applied Biosystems Step One Plus Real-Time system (Bio-Rad, USA). The thermal cycling conditions included an initial denaturation step at 95 °C for 10 min, followed by 40 cycles at 95 °C for 30 s, 60 °C for 30 s, and 72 °C for 30 s. Melting curve analysis of every qPCR was conducted after each cycle.

### Statistical analysis

Student’s *t* test, as implemented in Minitab 17 (Minitab Inc., State College, USA), two-way analysis of variance (ANOVA) was used to statistically evaluate the results. Data were presented as the mean ± standard deviation (SD). Statistical significance was set at *P* < 0.05.

### Reporting summary

Further information on research design is available in the Nature Research Reporting Summary linked to this article.

### Supplementary information

Reporting Summary

## Data Availability

The data that support the findings of this study are available from the corresponding author upon reasonable request.

## Code availability

Only public software was used to generate or process datasets in this work. The software version and websites were described in the “Methods” section. The underlying code including NCBI codes utilized to sequence primer is not publicly available but may be made available to qualified researchers on reasonable request from the corresponding author.
